# Comparison of the accuracy and efficacy of different assistive techniques in primary total knee arthroplasty: A network meta‐analysis

**DOI:** 10.1002/jeo2.70098

**Published:** 2024-11-28

**Authors:** Yuhang Zheng, Yang Li, Ziqi Yuan, Xiao Geng, Hua Tian

**Affiliations:** ^1^ Department of Orthopedics Peking University Third Hospital Beijing China; ^2^ Engineering Research Center of Bone and Joint Precision Medicine Ministry of Education Beijing China

**Keywords:** computer‐assisted navigation system, conventional cutting instrument, network meta‐analysis, patient‐specific instrument, robot‐assisted system, total knee arthroplasty

## Abstract

**Purpose:**

Various assistive techniques, such as conventional cutting instruments (CON), computer‐assisted navigation systems (CAS), patient‐specific instruments (PSI) and robot‐assisted systems (RAS), have been developed and applied in primary total knee arthroplasty (TKA). In this study, we aimed to assess the relative accuracy and efficacy of several assistive techniques for TKA through a network meta‐analysis (NMA) based on multiple published randomized controlled trials (RCTs).

**Methods:**

The PubMed, EMBASE and Cochrane databases were searched for RCTs to conduct this NMA from inception to 1 January 2024. We combined direct and indirect comparisons using a Bayesian NMA framework to assess and compare the effects of different assistive techniques on radiological and clinical outcomes. An NMA was conducted, and the study protocol was published online at PROSPERO (CRD42023402882).

**Results:**

One hundred and twelve RCTs involving 14,968 TKAs with four different assistive techniques (CON, CAS, PSI and RAS) were evaluated. Inconsistency and heterogeneity were acceptable for most outcomes. Based on the surface under the cumulative ranking curve, RAS could be the best technique for accurate mechanical axis alignment and component position, followed by CAS, PSI and CON. We observed no difference in clinical outcome scores. Additionally, CAS was the best intervention for visual analogue scale scores, and PSI had the shortest operative time. No significant differences were observed in postoperative complications, range of motion or total blood loss.

**Conclusion:**

RAS was most likely to achieve an accurate alignment, followed by CAS, PSI and CON. No differences were observed in clinical outcome scores and postoperative complications among the four assistive techniques.

**Level of Evidence:**

Level I (systematic review of Level‐I randomized controlled studies).

**Abbreviations**
CAS: computer‐assisted navigation systems
CI: confidence interval
CON: conventional cutting instruments
KSFS: Knee Society Functional Score
KSKS: Knee Society Knee Score
MD: mean difference
NMA: network meta‐analysis
OKS: Oxford knee score
PSI: patient‐specific instruments
RAS: robot‐assisted systems
RCT: randomized controlled trial
ROM: range of motion
RR: relative risks
SUCRA: the surface under the cumulative ranking curve
TKA: total knee arthroplasty
VAS: visual analogue scale
WOMAC: Western Ontario and McMaster Universities Osteoarthritis Index

## INTRODUCTION

Total knee arthroplasty (TKA) is one of the most effective therapeutic options for patients who have end‐stage knee osteoarthritis [52, 54]. Surgical methods and prosthesis placement accuracy can significantly influence the therapeutic effects of TKA and patient satisfaction [109]. Conventional cutting instruments (CON) are challenging and lack precise alignment [58]. To improve the accuracy of prosthesis placement and lower limb alignment and improve patient satisfaction, novel assistive techniques, including patient‐specific instruments (PSI), computer‐assisted navigation systems (CAS) and robot‐assisted systems (RAS), are applied to TKA.

Over the past 20 years, many scholars have compared the therapeutic effects of these novel assistive techniques and CON; however, the current research results remain controversial [9, 21, 51, 84, 134, 153]. These interventions (CAS, PSI and RAS) are precise in osteotomy, alignment and prosthesis positioning [16, 74] and reduce iatrogenic bone and periarticular soft tissue damage [61, 75, 91]. However, they may increase radiation exposure and medical care costs. They have been associated with unique complications such as pin tract‐induced fractures [6, 121], and their efficacy in postoperative clinical outcome scores remains unclear.

Furthermore, conventional meta‐analyses of the therapeutic effects of novel techniques have been conducted on effect size based on pairwise head‐to‐head direct comparisons; however, data from direct comparisons are relatively limited [32]. Network meta‐analysis (NMA) can perform direct comparisons and indirect comparisons based on logical inference, even though the two interventions have never been compared directly [11, 17, 32, 55].

We conducted an NMA of randomized controlled trials (RCTs) to comprehensively evaluate the relative accuracy and efficacy of different assistive techniques and to provide an objective rank of various interventions based on the corresponding surface under the cumulative ranking curve (SUCRA). We hypothesized that CAS, PSI and RAS would improve the accuracy of alignment and clinical outcomes compared to CON.

## METHODS

### Information sources and search strategy

A comprehensive search was conducted in three databases (PubMed, EMBASE and Cochrane Library) from their inception to 1 January 2024, using a combination of MeSH terms and free words. Key terms included diseases, treatments and study types, such as ‘total knee arthroplasty’, ‘computer‐assisted navigation’, ‘robotics’, ‘patient‐specific instrumentation’ and ‘randomized controlled trials’, and their synonyms were searched. Additional eligible studies were collected from the reference lists of some retrieved publications or relevant meta‐analyses. Two investigators independently performed the search. This NMA of RCTs was reported in accordance with the Preferred Reporting Items for Systematic Review and Meta‐Analysis statement extension for NMA [102]. And this study was registered at PROSPERO (registration number CRD42023402882).

### Types of outcome measures

- Radiological outcomes: (1) mechanical axis outliers; (2) coronal femoral component angle outliers; (3) coronal tibial component angle outliers; (4) sagittal femoral component angle outliers and (5) sagittal tibial component angle outliers. Outliers were defined as deviations ≥3° from the neutral alignment.
- Clinical outcomes: (1) short‐term Knee Society Knee Score (KSKS); (2) short‐term Knee Society Function Score (KSFS); (3) short‐term Western Ontario and McMaster Universities Osteoarthritis Index (WOMAC) scores; (4) short‐term Oxford knee score (OKS); (5) medium‐and‐long‐term KSKS; (6) medium‐and‐long‐term KSFS; (7) medium‐and‐long‐term WOMAC scores; (8) medium‐and‐long‐term OKS; (9) postoperative complications; (10) range of motion (ROM); (11) visual analogue scale (VAS) scores; (12) operative time and (13) total blood loss. Short‐term was defined as a follow‐up period of ≤2 years and medium‐and‐long‐term was defined as a follow‐up period of >2 years.

### Data extraction and quality assessment

Two researchers (Z. Y. H. and Y. Z. Q.) independently reviewed the titles and abstracts of the articles retrieved from the literature search to determine whether a study was eligible for inclusion. If we encountered the problem of missing data, we would attempt to contact the original authors. The reasons for exclusion are detailed in the flow chart. For each study, two researchers independently extracted data on general study information, participant demographics, type of assistive technique and outcomes of interest.

Two independent researchers (Z. Y. H. and Y. Z. Q.) assessed the quality of the included studies using the Cochrane Risk of Bias tool [43]. Any disagreements were resolved through consultation between the two researchers or by a third researcher (L. Y.). Review Manager software (version 5.3.5) was used for the quality assessment.

### Data analyses

We performed pairwise meta‐analyses by synthesizing studies that compared the same interventions to incorporate the assumption that different studies assessed different treatment effects. The forest plots and *I* ^2^ statistics were visually inspected to investigate the possibility of statistical heterogeneity. *I* ^2^ > 50% indicates significant heterogeneity.

Statistical significance tests were performed for global and local loop inconsistency using a global chi‐square test and network node‐splitting method, respectively, whereby a *p *< 0.05 may indicate significant inconsistency [44, 119]. Bayesian NMA was used to analyze the indirect comparison results of the assistive technologies. Mean difference (MD) and relative risks (RR) were used to calculate continuous and binary outcomes, respectively. A 95% confidence interval (CI) for each outcome was calculated, which is significant for those yielding a 95% CI of RR, excluding 1, or MD, excluding 0.

The SUCRA was drawn to calculate the cumulative ranking probability of each assistive technique scheme based on the area under the curve [113, 137]. The higher the SUCRA value, the better the effect of the assistive technique. Funnel plots were used to reflect the publication bias of the outcomes. Stata software (version 17.0; StataCorp LLC) was used for the statistical analysis.

## RESULTS

### Study selection and characteristics

**Figure 1:**
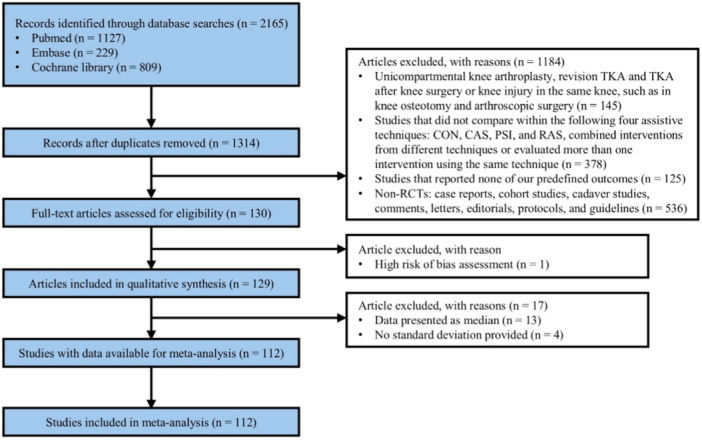
Flowchart for selection of included studies. CAS, computer‐assisted navigation systems; CON, conventional cutting instruments; PSI, patient‐specific instruments; RAS, robot‐assisted systems.

Overall, 2016 relevant studies were obtained in the initial search of the PubMed (*n* = 1127), Embase (*n* = 229) and Cochrane Library (*n *= 809) databases. After excluding duplicate studies and applying the inclusion and exclusion criteria, a total of 112 RCTs with 14968 TKAs were included in this NMA (Figure 1).

**Figure 2:**
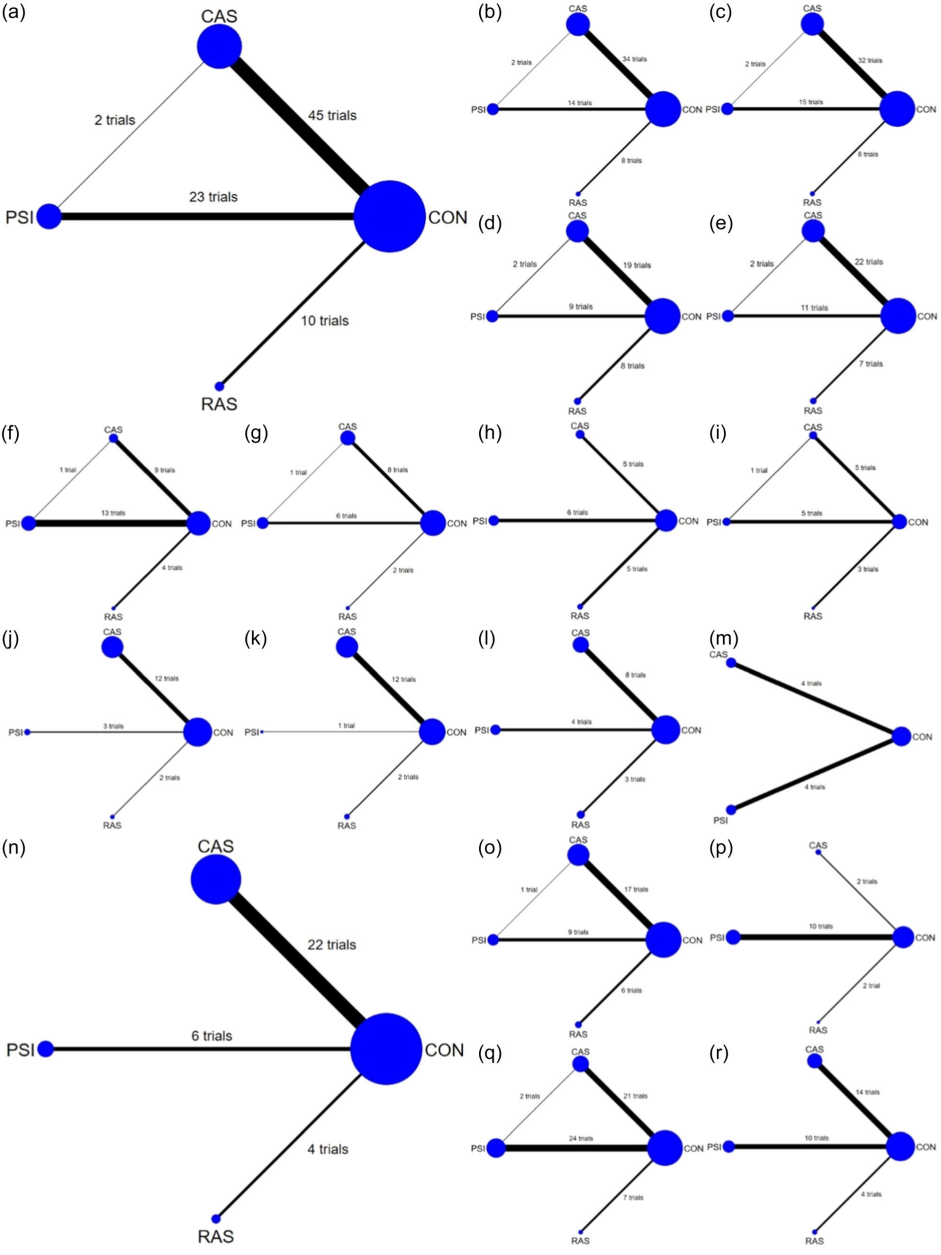
Network map of available comparisons for a network meta‐analysis of (a) mechanical axis outliers, (b) coronal femoral component angle outliers, (c) coronal tibial component angle outliers, (d) sagittal femoral component angle outliers, (e) sagittal tibial component angle outliers, (f) short‐term and (g) medium‐and‐long‐term Knee Society Knee Scores, (h) short‐term and (i) medium‐and‐long‐term Knee Society Function Scores, (j) short‐term and (k) medium‐and‐long‐term the Western Ontario and McMaster Universities scores, (l) short‐term and (m) medium‐and‐long‐term Oxford knee scores, (n) postoperative complications, (o) range of motion, (p) visual analogue scale scores, (q) operative time and (r) total blood loss. The size of the nodes indicates the number of TKAs and the thickness of the lines indicates the number of direct comparisons between different assistive techniques. CAS, computer‐assisted navigation systems; CON, conventional cutting instruments; RAS, robot‐assisted systems.

The available direct comparisons of the studies and network map are shown in Figure 2. One three‐arm trial [149] that compared CON, CAS and PSI was included in the analysis, whereas the remainder was two‐arm trials. CAS was compared with CON in 59 trials [4, 7, 12, 19, 20, 23, 24, 26, 30, 31, 33, 34, 37, 38, 41, 45, 46, 48, 53, 56, 62, 63, 64, 65, 66, 68, 72, 82, 85, 86, 87, 88, 90, 93, 99, 100, 101, 103, 104, 105, 116, 117, 118, 122, 126, 127, 128, 135, 139, 140, 145, 148, 150, 152, 154, 155, 156, 157, 158] and with PSI in one trial [144]. Thirty‐six trials [1, 2, 5, 14, 15, 18, 25, 28, 35, 39, 40, 49, 50, 69, 70, 71, 76, 77, 94, 95, 98, 106, 108, 110, 112, 114, 115, 125, 129, 131, 133, 141, 142, 143, 146] compared PSI with CON and 15 trials [13, 22, 27, 67, 78, 79, 80, 81, 123, 124, 132, 136, 138, 147, 151] compared RAS and CON. However, none of the eligible studies directly compared RAS with CAS or PSI. The basic characteristics of the included studies are presented in Table 1, and detailed characteristics are shown in Supporting Information S1: Supplement 2.

**Table 1:** Assistive techniques' demographic characteristics.

Assistive technique	No. of groups	No. of TKAs	Age (year)	Women/total	BMI (kg/m^2^)	Year first RCT published
CON	111	7404	66.8	0.74	28.5	2003
CAS	61	4476	67.1	0.76	28.2	2003
PSI	38	1615	68.2	0.68	29.2	2013
RAS	15	1473	63.2	0.77	27.6	2011

*Note*: Age and BMI are presented as mean.

Abbreviations: BMI, body mass index; CAS, computer‐assisted navigation systems; CON, conventional cutting instruments; PSI, patient‐specific instruments; RAS, robot‐assisted systems; RCT, randomized controlled trial; TKA, total knee arthroplasty.

### Risk of bias assessment

**Figure 3:**
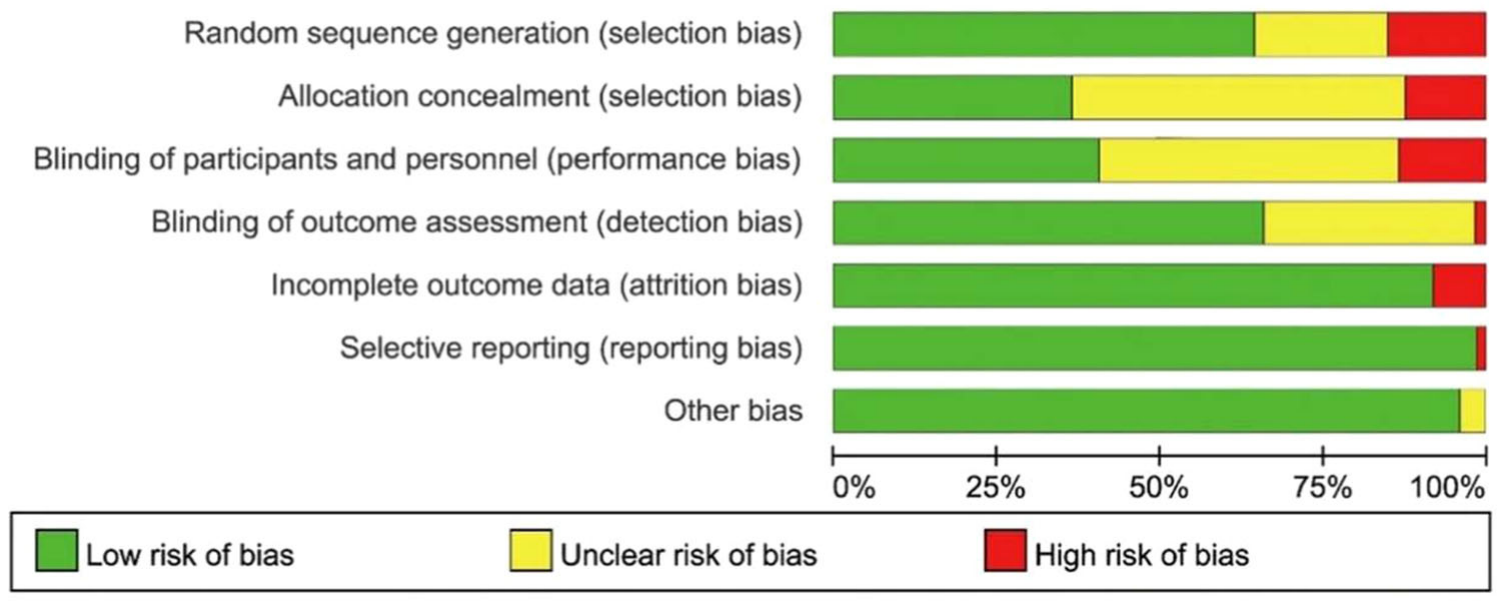
Risk of bias assessment: overall risk of bias for all trials included.

The results of the bias risk assessment for the 113 studies are presented in Figure 3. All of these studies were randomized trials. Seventeen studies (15%) had a high risk of bias in random sequence generation, while fourteen studies (13%) had a high risk of bias in allocation concealment. Additionally, 15 studies (13%) were at high risk of performance bias, and two studies (2%) faced a high risk of bias in blinding of outcome assessment. Furthermore, nine studies (8%) had incomplete outcome data. Notably, one study was excluded from the final data analysis due to a high risk of reporting bias.

### Radiological outcomes

#### Mechanical axis

**Figure 4:**
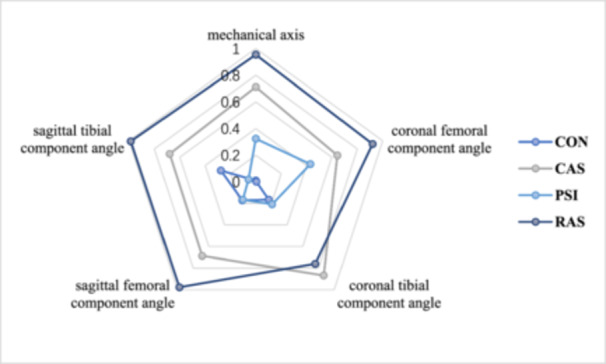
Value of SUCRA of radiological outcomes. CAS, computer‐assisted navigation systems; CON, conventional cutting instruments; PSI, patient‐specific instruments; RAS, robot‐assisted systems; SUCRA, surface under the cumulative ranking curve.

Seventy‐eight studies assessed mechanical axis outliers and were eligible for NMA. The proportion of mechanical axis outliers was lower in the CAS group (vs. CON, RR: 0.41, 95% CI: 0.33–0.50; vs. PSI, RR: 0.63, 95% CI: 0.48–0.83, respectively) and in the RAS group (vs. CON, RR: 0.40, 95% CI: 0.28–0.59; vs. PSI, RR: 0.51, 95% CI: 0.33–0.78, respectively), with significant differences (*p* < 0.05) (Table 2). The treatment rankings based on SUCRA scores, from largest to smallest, were RAS (95.4%), CAS (71.2%), PSI (32.4%) and CON (1%) (Figure 4).

**Table 2:** RR and corresponding 95% confidence intervals for radiological outcomes.

Assistive technique	Mechanical axis outliers (RR)	Coronal femoral component angle outliers (RR)	Coronal tibial component angle outliers (RR)	Sagittal femoral component angle outliers (RR)	Sagittal tibial component angle outliers (RR)
RAS vs.					
PSI	**0.51 (0.33, 0.78)**	0.66 (0.36, 1.18)	0.58 (0.27, 1.25)	**0.42 (0.24, 0.75)**	**0.26 (0.13, 0.52)**
CAS	0.80 (0.54, 1.19)	0.76 (0.45, 1.29)	1.10 (0.55, 2.20)	0.68 (0.40, 1.15)	0.58 (0.31, 1.08)
CON	**0.40 (0.28, 0.59)**	**0.44 (0.27, 0.72)**	0.56 (0.30, 1.05)	**0.43 (0.27, 0.68)**	**0.32 (0.18, 0.55)**
PSI vs.					
CAS	**1.58 (1.20, 3.07)**	1.17 (0.78, 1.74)	**1.90 (1.10, 3.28)**	**1.60 (1.04, 2.47)**	**2.22 (1.36, 3.64)**
CON	0.80 (0.63, 1.00)	**0.68 (0.48, 0.95)**	**0.97 (0.38, 0.69)**	1.01 (0.72, 1.42)	1.21 (0.81, 1.80)
CAS vs.					
CON	**0.50 (0.43, 0.60)**	**0.58 (0.46, 0.73)**	**0.51 (0.38, 0.69)**	**0.63 (0.48, 0.83)**	**0.54 (0.40, 0.73)**

*Note*: Results with statistically significant differences have been highlighted in bold.

Abbreviations: CAS, computer‐assisted navigation systems; CON, conventional cutting instruments; PSI, patient‐specific instruments; RAS, robot‐assisted systems; RR, relative risks.

#### Component position accuracy

The NMA results for coronal femoral component angle outliers, coronal tibial component angle outliers, sagittal femoral component angle outliers and sagittal tibial component angle outliers are shown in Table 2, and the SUCRA values for these outcomes are illustrated in Figure 4 and Supporting Information S1: Supplement 4.

We observed that compared to CON, CAS (RR: 0.58, 95% CI: 0.46–0.73), PSI (RR: 0.68, 95% CI: 0.48–0.95) and RAS (RR: 0.44, 95% CI: 0.27–0.72) reduced the proportion of coronal femoral component angle outliers. Regarding coronal tibial component angle outliers, the CAS group reduced the proportion of outliers compared with CON (RR: 0.51, 95% CI: 0.38–0.69) and compared with PSI (RR: 0.53, 95% CI: 0.30–0.91). The proportions of sagittal femoral component angle outliers and sagittal tibial component angle outliers were significantly lower in the CAS and RAS groups (*p* < 0.05).

The RAS group showed the lowest proportion of coronal femoral component angle outliers, sagittal femoral component angle outliers, and sagittal tibial component angle outliers with SUCRA values of 92%, 97.6% and 98.6%, respectively. Moreover, the CAS group ranked first regarding coronal tibial component angle outliers, with SUCRA values of 86.7%. SUCRA indicated that RAS was the ideal intervention for the component position.

### Clinical outcomes

#### Clinical outcome scores

The NMA results for the clinical outcome scores are shown in Tables 3 and 4. The NMA results showed no significant differences in either short‐term or medium‐and‐long‐term clinical outcome scores between the different assistive techniques (*p* > 0.05).

**Table 3:** MD and corresponding 95% confidence intervals for short‐term clinical outcome scores.

Assistive technique	Short‐term KSKS (MD)	Short‐term KSFS (MD)	Short‐term WOMAC scores (MD)	Short‐term OKS scores (MD)
RAS vs.				
PSI	−0.71 (−3.67, 2.25)	0.52 (−5.29, 6.33)	−0.01 (−3.85, 3.82)	−0.57 (−3.81, 2.67)
CAS	0.41 (−2.51, 3.34)	−1.74 (−7.04, 3.57)	0.85 (−2.99, 4.68)	0.36 (−2.78, 3.50)
CON	0.17 (−2.38, 2.72)	−0.03 (−4.73, 4.66)	0.25 (−3.40, 3.90)	−0.57 (−3.81, 2.67)
PSI vs.				
CAS	1.12 (−0.82, 3.07)	−2.26 (−6.29, 1.78)	0.86 (−0.82, 2.54)	0.93 (−1.52, 3.38)
CON	0.88 (−0.55, 2.31)	−0.55 (−3.96, 2.85)	0.26 (−0.93, 1.45)	0.95 (−0.96, 2.87)
CAS vs.				
CON	−0.24 (−1.66, 1.17)	1.71 (−0.72, 4.13)	−0.60 (−1.78, 0.59)	0.02 (−1.72, 1.77)

Abbreviations: CAS, computer‐assisted navigation systems; CON, conventional cutting instruments; KSFS, Knee Society Functional Score; KSKS, Knee Society Knee Score; MD, mean difference; OKS, Oxford knee score; PSI, patient‐specific instruments; RAS, robot‐assisted systems; WOMAC, Western Ontario and McMaster Universities Osteoarthritis Index.

**Table 4:** MD and corresponding 95% confidence intervals for medium‐and‐long‐term clinical outcome scores.

Assistive technique	Medium‐and‐long‐term KSKS (MD)	Medium‐and‐long‐term KSFS scores (MD)	Medium‐and‐long‐term WOMAC scores (MD)	Medium‐and‐long‐term OKS scores (MD)
RAS vs.				
PSI	−0.42 (−5.15, 4.31)	0.66(−2.93, 4.25)	−0.95 (−4.58, 2.68)	
CAS	0.44 (−4.30, 5.17)	−1.54 (−3.97, 0.89)	0.02 (−3.56, 3.53)	
CON	−0.16 (−4.74, 4.42)	−1.51 (−3.46, 0.43)	0.01 (−3.05, 3.06)	
PSI vs.				
CAS	0.86 (−4.30, 5.17)	−2.20 (−5.71, 1.32)	0.93 (−1.56, 3.44)	0.37 (−3.89, 3.14)
CON	0.26(−0.93, 1.45)	−2.17 (−5.34, 1.00)	0.96 (−1.01, 2.92)	−0.31 (−2.89, 2.27)
CAS vs.				
CON	−0.60 (−1.76, 0.59)	0.03 (−1.42, 1.48)	0.02 (−1.77, 1.82)	0.06 (−2.22, 2.34)

Abbreviations: CAS, computer‐assisted navigation systems; CON, conventional cutting instruments; KSFS, Knee Society Functional Score; KSKS, Knee Society Knee Score; MD, mean difference; OKS, Oxford knee score; PSI, patient‐specific instruments; RAS, robot‐assisted systems; WOMAC, Western Ontario and McMaster Universities Osteoarthritis Index.

**Figure 5:**
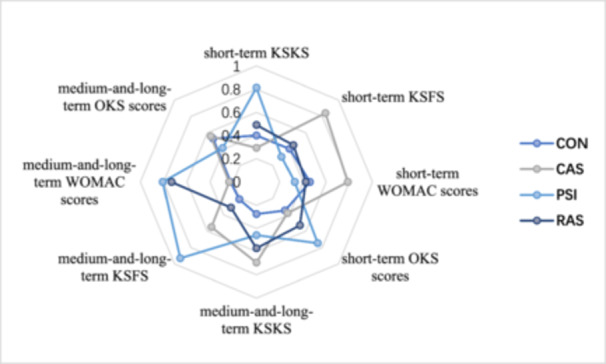
Value of SUCRA of clinical outcome scores. CAS, computer‐assisted navigation systems; CON, conventional cutting instruments; KSFS, Knee Society Function Score; KSKS, Knee Society Knee Score; OKS, Oxford knee score; PSI, patient‐specific instruments; RAS, robot‐assisted systems; SUCRA, surface under the cumulative ranking curve; WOMAC, the Western Ontario and McMaster Universities.

Through ranking analysis based on SUCRA values, as shown in Figure 5 and Supporting Information S1: Supplement 4, the PSI group showed the best score in short‐term KSKS, short‐term OKS, medium‐and‐long‐term KSFS and medium‐and‐long‐term WOMAC with SUCRA values of 81.3%, 74.6%, 93.0% and 80.8%, respectively. The CAS group ranked first in terms of short‐term KSFS, short‐term WOMAC, medium‐and‐long‐term KSKS and medium‐and‐long‐term OKS with SUCRA values of 84.0%, 78.7%, 69.5% and 55.8%.

#### Postoperative complications

Thirty‐two studies assessed postoperative complications and were eligible for the NMA (Table 5). The NMA results showed no significant differences in the incidence of postoperative complications among the different assistive techniques (*p* > 0.05). The treatment rankings based on the SUCRA scores, from largest to smallest, were as follows: RAS (78.9%), CAS (69.1%), CON (27.2%) and PSI (24.8%) (Table 6).

**Table 5:** RR or MDs and corresponding 95% confidence intervals for clinical outcomes, excluding clinical outcome scores.

Assistive technique	Postoperative complications (RR)	Range of motion (MD)	Visual analogue scale scores (MD)	Operative time (MD)	Total blood loss (MD)
RAS vs.					
PSI	0.73 (0.39, 1.37)	−0.52 (−3.01, 1.97)	0.45 (−0.20, 1.10)	**30.19 (19.92, 40.47)**	−1.79 (−90.92, 87.34)
CAS	0.89 (0.53, 1.51)	−0.45 (−2.55, 1.64)	**0.88 (0.04, 1.72)**	**11.78 (1.36, 22.20)**	−31.40 (−118.19, 55.39)
CON	0.77 (0.47, 1.24)	0.08 (−1.73, 1.90)	0.19 (−0.40, 0.77)	**24.84 (15.76, 33.91)**	−49.04 (−129.62, 31.53)
PSI vs.					
CAS	1.23 (0.77, 1.95)	0.06 (−1.93, 2.06)	0.43 (−0.23, 1.09)	**−18.41 (−25.19, −11.63)**	−29.61 (−85.36, 26.14)
CON	1.05 (0.70, 1.59)	0.60 (−1.08, 2.28)	−0.26 (−0.54, 0.02)	**−5.36 (−10.19, −0.53)**	−47.25 (−87.92, −6.59)
CAS vs.					
CON	0.86 (0.70, 1.06)	0.53 (−0.54, 1.61)	**−0.69 (−1.29, −0.09)**	**13.05 (7.93, 18.18)**	−17.64 (−56.16, 20.88)

*Note*: Results with statistically significant differences have been highlighted in bold.

Abbreviations: CAS, computer‐assisted navigation systems; CON, conventional cutting instruments; MD, mean difference; PSI, patient‐specific instruments; RAS, robot‐assisted systems; RR, relative risks.

**Table 6:** Value of SUCRA of postoperative complications, ROM, VAS scores, operative time and total blood loss.

Assistive technique	Postoperative complications	ROM	VAS scores	Operative time	Total blood loss
CON	0.272	0.287	0.262	**0.671**	0.104
CAS	**0.691**	**0.664**	**0.958**	0.328	0.399
PSI	0.248	**0.649**	**0.658**	**0.995**	**0.774**
RAS	**0.789**	0.399	0.122	0.005	**0.723**

*Note*: The fonts for the two assistive techniques with higher SUCRA values have been bolded.

Abbreviations: CAS, computer‐assisted navigation systems; CON, conventional cutting instruments; PSI, patient‐specific instruments; RAS, robot‐assisted systems; ROM, range of motion; SUCRA, the surface under the cumulative ranking curve; VAS, visual analogue scale.

#### ROM

The ROM data at the last follow‐up after TKA was extracted from 31 studies. No significant differences were observed among the different assistive techniques. The results of the pairwise meta‐analysis are shown in Table 5. The rankings based on SUCRA scores were as follows: CAS (66.4%), PSI (24.8%), RAS (39.9%) and CON (28.7%).

#### VAS score

The VAS scores at the last follow‐up after TKA were extracted from 14 studies. The VAS score of the CAS group was lower than that of the CON group (MD: −0.69, 95% CI: −1.29 to −0.09) and the RAS group (MD: −0.88, 95% CI: −1.72 to −0.04). The CAS group had the highest SUCRA value (95.8%), whereas the RAS group had the minimum value (12.2%).

#### Operative time

Fifty‐two studies were included in the analysis. The results of the pairwise meta‐analysis are shown in Table 5. The operative time was significantly shorter in the PSI group than in the other groups (*p* < 0.05). The time in the CON group was significantly shorter than that in the CAS and RAS groups (*p* < 0.05). The time of the CAS group was shorter than that of the RAS group (MD: −11.78, 95% CI: −22.20 to −1.36). Data from the pairwise meta‐analysis provided evidence of significant heterogeneity (minimum *I* ^2^ = 77.4%, maximum *I* ^2^ = 98.7%; Supporting Information S1: Supplement 3).

#### Total blood loss

Data on total blood loss was extracted from 28 studies. The NMA results showed no difference in total blood loss except for PSI, which showed a significant difference compared with CON. Data from the pairwise meta‐analysis provided evidence of significant heterogeneity (minimum *I* ^2^ = 69.6%, maximum *I* ^2^ = 94.7%; Supporting Information S1: Supplement 3).

## DISCUSSION

New assistive techniques are a popular topic in the TKA field. The present meta‐analysis and systematic review showed that each assistive technique has advantages and disadvantages. Our NMA of alignment accuracy and clinical outcomes summarizes worldwide efforts during the last two decades to identify optimal assistive techniques. The main finding of this NMA is that RAS provides the greatest advantage in achieving accurate alignment, followed by CAS, PSI and CON, but the clinical outcomes among these four techniques were similar.

### Discussion of assistive techniques for radiological outcomes

The accuracy of the mechanical axis alignment and component position in TKA is an important factor to improve knee function, patient satisfaction and prosthesis survival [58, 92]. A deviation of ±3° from the neutral alignment was acceptable, and a deviation beyond this range predisposed the patient to early implant failure and reduced implant survival [10, 57, 130]. Our analysis showed that the rates of postoperative mechanical axis alignment inliers in the CAS (89%) and RAS (89%) groups were significantly higher than those in the CON (78%) and PSI (81%) groups. An NMA that compared RAS, CAS and CON found that both RAS and CAS could improve mechanical axis alignment [36]. However, another NMA indicated that approximately 10% more patients achieved mechanical axis alignment inliers with CAS compared to CON or PSI [16]. This study did not identify any advantage of RAS over CON or PSI in achieving mechanical axis alignment inliers.

Regarding the component position, we observed that the RAS and CAS groups achieved better femoral and tibial component alignment than the PSI and CON groups. The latest NMA by Lei et al. [74], which also compared alignment and component position, reached similar outcomes. The accuracy of radiological alignment is believed to correlate with postoperative complications. RAS and CAS can improve alignment; however, the thresholds necessary to achieve better outcomes and survival remain unclear.

All included studies used mechanical alignment as the standard for both surgical and postoperative evaluation. Kinematic alignment and functional alignment are better suited for reconstructing the natural kinematic characteristics of the knee joint and reducing the need for soft tissue release around the knee [42, 47, 97]. This could represent a potential advantage of RAS and CAS. By facilitating more precise osteotomy and alignment, these methods enable a more accurate and reproducible attainment of nonneutral alignment targets. This reduces the risk of significant deviation from the target value postoperatively and better facilitates kinematic and functional alignment.

### Discussion of assistive techniques on clinical outcome scores

The most common clinical outcomes measured in the included studies were KSKS, KSFS, WOMAC and OKS scores. This NMA showed no significant differences in clinical outcome scores after TKA assisted by the four techniques. This finding is consistent with those of the present NMA by Lei et al. [74] and Bouché et al. [16], who determined that the above four techniques showed no clinical significance in postoperative outcomes. However, this differs from the findings of other meta‐analyses. Agarwal [3] reported that significance was noted postoperatively in favour of RAS compared to CON for TKA when considering the WOMAC and HSS scores. Rudran [111] reported that PSI improved KSKS, KSFS and WOMAC scores at 2 years compared with CON.

The significance of the clinical outcome scores of the above studies did not reach the minimal clinically important difference (MCID). The MCID values within the literature for the OKS, KSKS and KSFS scores were 4.7–10, 5.3–9 and 5.0–8.1, respectively [73, 83, 89]. A wide range of MCID values for the WOMAC subscales have been reported, ranging from 20.5–36.0 for pain, 17.6–33.0 for function and 12.9–25.0 for stiffness [89]. Thus, no differences were observed among the four assistive techniques.

The benefits observed in alignment and component positioning did not translate into improvements in clinical outcome scores. This may be due to the reference system relying on bony landmarks and not accounting for soft tissue balancing, which is a key factor influencing clinical outcomes.

### Discussion of assistive techniques on operation‐related indexes

RAS and CAS required longer operative time than CON and PSI; however, RAS and CAS had better advantages in terms of postoperative complications, according to the SUCRA statistics. This may be because RAS and CAS can improve radiological alignment, thus reducing complications and revisions due to malalignment [8, 134]. Furthermore, although RAS and CAS significantly extend the duration of surgery, this additional time is predominantly spent on preoperative planning and intraoperative registration. Consequently, the most traumatic phases, such as joint capsule opening, blood vessel exposure or the intramedullary canal of the femur, remain unaffected by these extended durations. Notably, no complications that might be specifically related to robotic or navigation assistance, such as pin site fractures and pin tract infections, were reported in the included studies. The operative time in the PSI group was shorter than that in the other groups because of the simplified operative procedures [75].

According to the statistical results of SUCRA, CAS may be the best option to improve postoperative VAS scores. Few studies have evaluated VAS scores after using CAS [12, 118] or RAS [138, 151], and the VAS score was the only index reported sufficiently often to be included in our analysis, making it challenging to assess the effects of pain relief after TKA.

No significant difference was observed in total blood loss between the four techniques except in the PSI group compared to the CON group. Blood loss in conventional TKA occurs when the intramedullary canal of the femur, excessive bone resection and iatrogenic soft‐tissue trauma are breached [59, 60, 96]. A tourniquet is routinely used in TKA, and the femoral canal is closed with cement or a bone plug before tourniquet release, thus diminishing the impact of femoral canal instrumentation [107]. Moreover, the increased duration of surgery and tourniquet use in TKA with computer‐assisted and RAS may be potential causes of additional blood loss [29, 120].

### Limitation

This study has some notable limitations. First, our results are limited by the quality of the available studies. Several of the included studies were assessed as having a potentially high risk of bias. Second, the heterogeneity in some outcomes may have affected the results. We attempted to conduct a subgroup analysis to relieve these effects; however, we failed because the data were lacking. Third, this NMA lacked other outcomes, such as postoperative patient satisfaction, quality of life and implant longevity, because the data from the included RCTs were insufficient and heterogeneous. Fourth, all assistive techniques may offer potential benefits in achieving personalized alignment strategies. However, the included studies currently lack investigation into kinematic and functional alignment as alignment principles.

## CONCLUSION

This study revealed that RAS had the highest probability of achieving an accurate alignment, followed by CAS, PSI and CON. However, RAS and PSI had the longest and shortest time, respectively. No difference was observed among the four assistive techniques in most clinical outcomes, including short‐term or medium‐and‐long‐term clinical outcome scores and postoperative complications. The best assistive technique for a given outcome is not necessarily the best for others. Thus, the appropriate assistive technique should be selected based on the patient's perspective and clinical practice while considering the economic burdens of different techniques.

## AUTHOR CONTRIBUTIONS

**Hua Tian**: Conceptualization; supervision. **Yang Li**: Conceptualization; formal analysis; writing—review and editing. **Yuhang Zheng**: Methodology; formal analysis; writing—original draft; visualization. **Ziqi Yuan**: Methodology. **Xiao Gen**g: Writing—review and editing. All authors had full access to the data in the study and take responsibility for the integrity of the data and the accuracy of the data analysis.

## CONFLICT OF INTEREST STATEMENT

The authors declare no conflicts of interest.

## ETHICS STATEMENT

This study was registered at PROSPERO (registration number CRD42023402882).

## Supporting information

Supplementary Information

## Data Availability

The data sets analyzed during the current study are available from the corresponding author on reasonable request.
