# Supplementary material for: Comparison of the accuracy and efficacy of different assistive techniques in primary total knee arthroplasty: A network meta‐analysis
Source: J Exp Orthop. 2024 Nov 28;11(4):e70098. doi: 10.1002/jeo2.70098 (PMC11604599; doi:10.1002/jeo2.70098)
Supplement: Supplementary file 1 — Supplementary Information [file JEO2-11-e70098-s001.docx]

**Web appendix: Supplementary materials**

**Supplement 1: Each database retrieval strategy.**


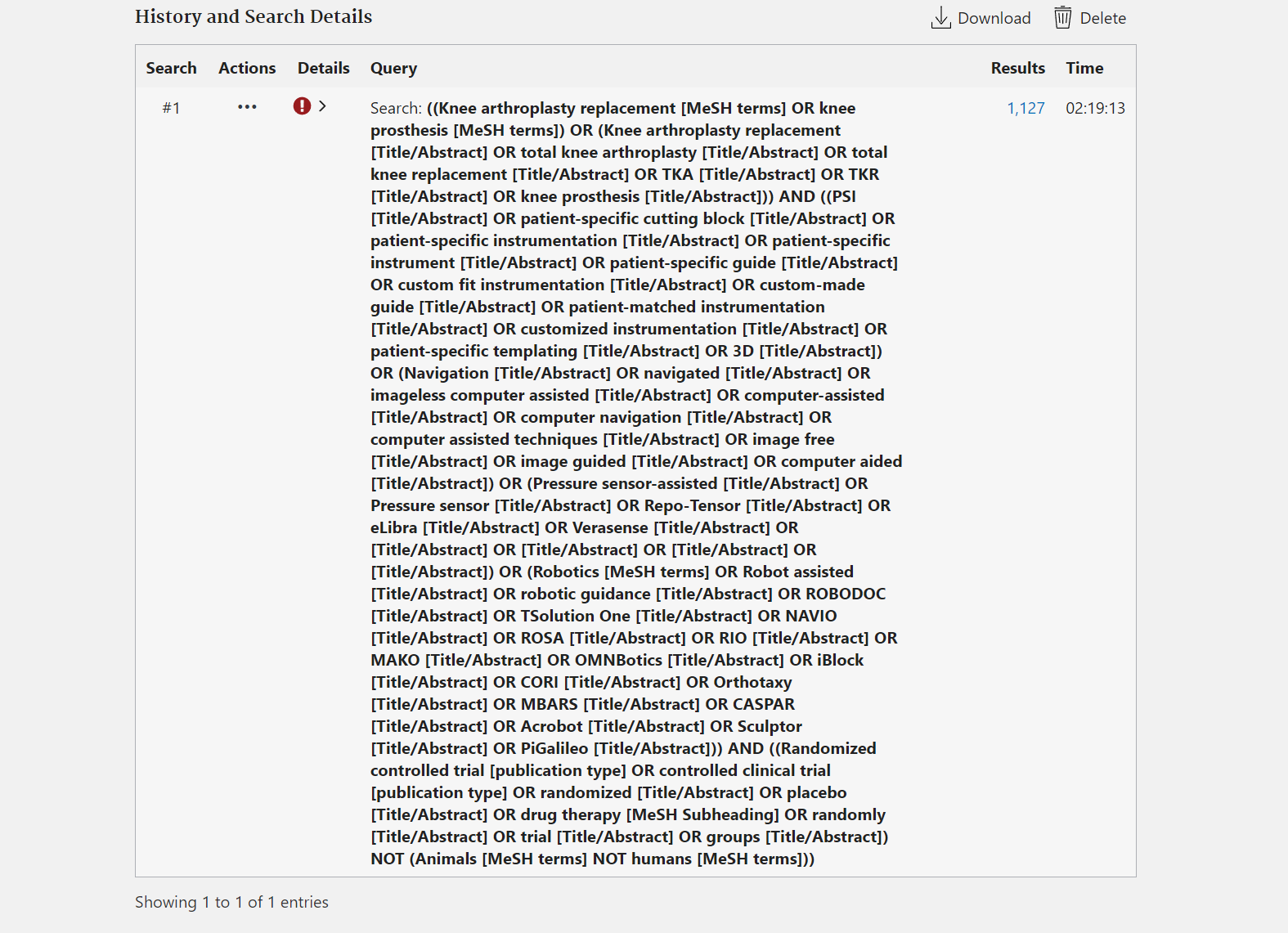


Fig. 1. Database retrieval strategy of PubMed


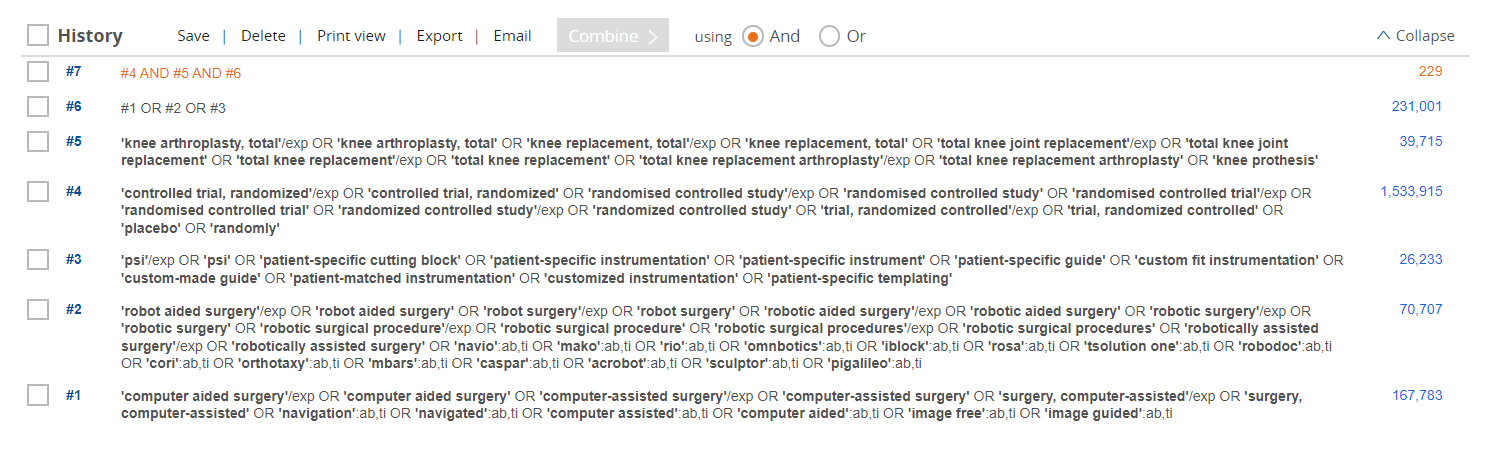


Fig. 2. Database retrieval strategy of EmBase


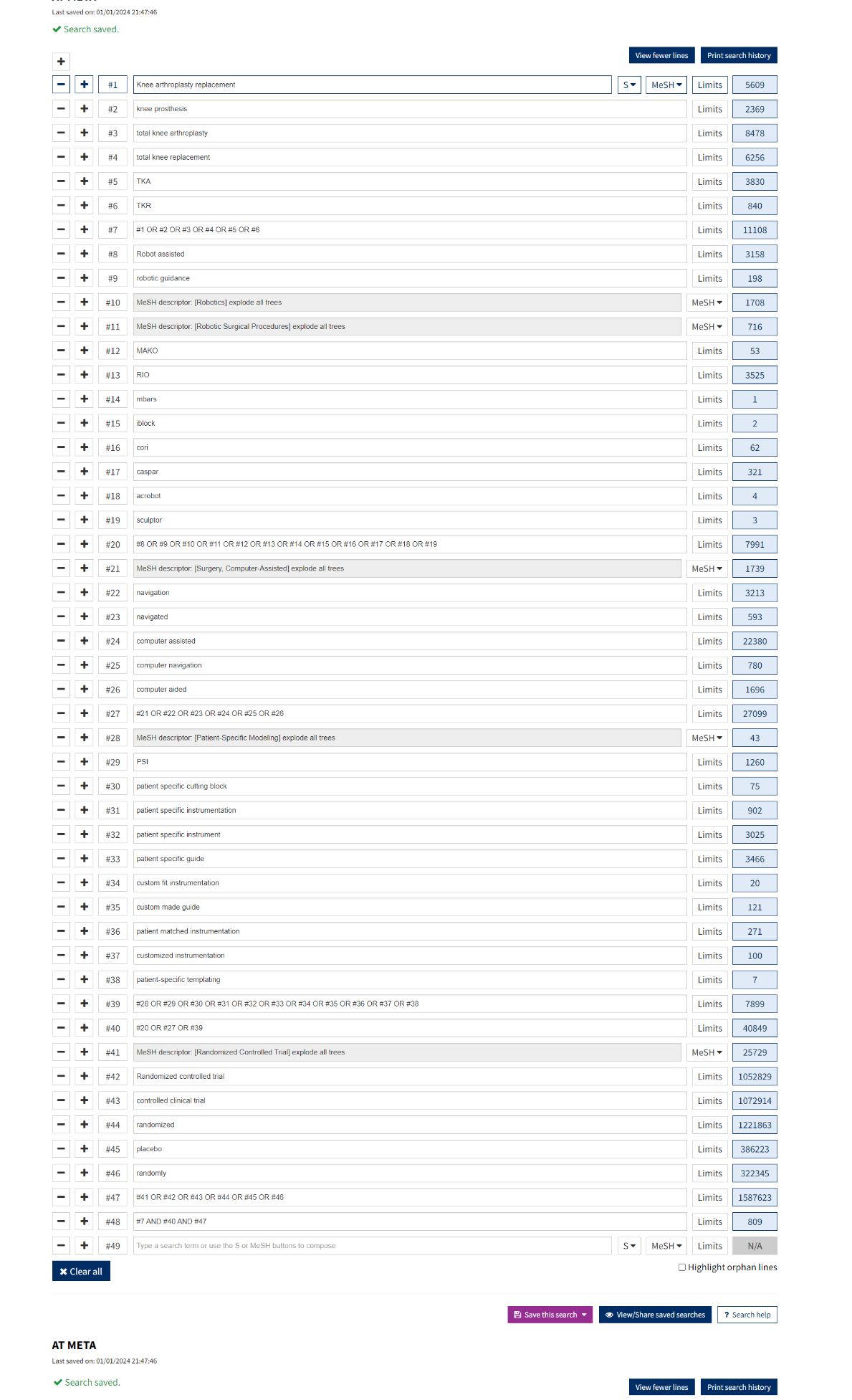


Fig. 3. Database retrieval strategy of Cochrane Library

**Supplement 2:** **Summary of characteristics and results of the included literature**

Table 1. Summary of characteristics and results of the included literature

|  | Authors | Year | Country | Assistive technique | Sample size | Type of RAS/CAS/PSI | Type of prosthesis | Patellar resurfacing | Mean follow-up | Radiographic outcomes | Clinical outcomes | Age (yr) | Gender (female/male) | BMI (kg/m2) |
| --- | --- | --- | --- | --- | --- | --- | --- | --- | --- | --- | --- | --- | --- | --- |
| 1 | Sparmann et al. | 2003 | Germany | CAS | 120 | Image-free knee navigation system (Stryker) | Duracon condylar TKA (Stryker) | NR | 3 months | a, b, c, d, e | n | 67.4 | 88/32 | NR |
|  |  |  |  | CON | 120 |  |  |  |  |  |  | 66.1 | 79/41 |  |
| 2 | Chuahan et al. | 2004 | Australia | CAS | 35 | Image-free knee navigation system (Stryker) | Duracon condylar TKA (Stryker) | No | 6 weeks | a, b, c | n | NR | NR | NR |
|  |  |  |  | CON | 35 |  |  |  |  |  |  |  |  |  |
| 3 | Stöckl et al. | 2004 | Austria | CAS | 32 | Image-free knee navigation system (Stryker) | Duracon condylar TKA (Stryker) | No | NA | a | - | 68.2±7.6 | 23/9 | NR |
|  |  |  |  | CON | 32 |  |  |  |  |  |  | 72.4±8.7 | 22/10 |  |
| 4 | Perlick et al. | 2004 | Germany | CAS | 50 | Image-free navigation system (BrainLab, Depuy) | NR | NR | NA | a, b, c | - | 66 | 40/10 | NR |
|  |  |  |  | CON | 50 |  |  |  |  |  |  | 72 | 42/8 |  |
| 5 | Decking et al. | 2005 | Germany | CAS | 27 | Image-free knee navigation system (OrthoPilot, Aesculap) | SEARCH Evolution, Aesculap (Tuttlingen) | Yes | 3 months | a, b, c, d, e | n, q, r | 64.7±9.4 | 18/9 | 27.9±3.55 |
|  |  |  |  | CON | 25 |  |  |  |  |  |  | 67.3±6.3 | 17/8 | 30.2±4.95 |
| 6 | Maculé-Beneyto  et al. | 2006 | Spain | CAS | 109 | Image-free knee navigation system (Stryker) | NR | NR | NA | a | - | 71.6 | NR | 30.4 |
|  |  |  |  | CON | 93 |  |  |  |  |  |  | 72.3 |  | 31.4 |
| 7 | Seon et al. | 2006 | Korea | CAS | 49 | Image-free knee navigation system (OrthoPilot, Aesculap) | NR | No | 2 weeks | a, b, c, e | n | 67.4 | 44/5 | NR |
|  |  |  |  | CON | 53 |  |  |  |  |  |  | 64.8 | 47/6 |  |
| 8 | Kim et al. | 2007 | Korea | CAS | 100 | Image-free navigation system (BrainLab, Depuy) | PFC Sigma (DePuy) | Yes | 2.3 years | a, b, c, d, e | j, k, n, q | 67.6±7.3 | 85/15 | 27.1±3.8 |
|  |  |  |  | CON | 100 |  |  |  |  |  |  | 67.6±7.3 | 85/15 | 27.1±3.8 |
| 9 | Mullaji et al. | 2007 | India | CAS | 282 | Image-free navigation system (BrainLab, Depuy) | PFC Sigma (DePuy) | Yes | NA | a | - | 65.5 | 215/67 | NR |
|  |  |  |  | CON | 185 |  |  |  |  |  |  | 65.9 | 143/42 |  |
| 10 | Spencer et al. | 2007 | Australia | CAS | 35 | Image-free knee navigation system (Stryker) | Duracon condylar TKA (Stryker) | No | 2 years | - | h, l, m | NR | NR | NR |
|  |  |  |  | CON | 35 |  |  |  |  |  |  |  |  |  |
| 11 | Martin et al. | 2007 | Australia | CAS | 100 | Image-free navigation system (BrainLab, Depuy) | NexGen CR and NexGen LPS-flex (Zimmer) | NR | 3 months | a, b, c, e | o, q, r | 70.3±8.2 | 68/32 | 30.2±4.8 |
|  |  |  |  | CON | 100 |  |  |  |  |  |  | 71.1±7.5 | 73/27 | 28.2±4.6 |
| 12 | Matziolis et al. | 2007 | Germany | CAS | 32 | Image-free knee navigation system (PiGalileo) | TC-PLUS (Endoplus) | NR | 6 months | a, b, c, d, e | o, q, r | 71±7 | NR | 30.5±4.7 |
|  |  |  |  | CON | 28 |  |  |  |  |  |  | 70±9 |  | 31.7±6.4 |
| 13 | Chotanaphuti  et al. | 2008 | Thailand | CAS | 86 | Image-free navigation system (BrainLab, Depuy) | PFC PS implants (DePuy) | No | NA | a, b, c, d, e | r | 67.2±6.46 | 83/11 | NR |
|  |  |  |  | CON | 94 |  |  |  |  |  |  | 67.8±7.14 | 80/6 |  |
| 14 | Lützner et al. | 2008 | Germany | CAS | 40 | Image-free knee navigation system (Stryker) | Scorpio PS System (Stryker) | No | NA | a, b, c | n | 69 | 27/13 | 30.4 |
|  |  |  |  | CON | 40 |  |  |  |  |  |  | 69 | 24/16 | 29.4 |
| 15 | Dutton et al. | 2008 | Singapore | CAS | 52 | Image-free navigation system (BrainLab, Depuy) | PFC Sigma (DePuy) | Yes | 1 month | a, b, c, d, e | n | 68 | 44/8 | 27.6 |
|  |  |  |  | CON | 56 |  |  |  |  |  |  | 67 | 44/12 | 27.1 |
| 16 | Oberst et al. | 2008 | Germany | CAS | 34 | Image-free navigation system (BrainLab, Depuy) | LCS complete knee prosthesis (DePuy/Johnson&Johnson) | NR | 1.3 years | a | - | NR | NR | NR |
|  |  |  |  | CON | 35 |  |  |  |  |  |  |  |  |  |
| 17 | Weng et al. | 2009 | China | CAS | 60 | Image-free navigation system (BrainLab, Depuy) | PFC Sigma (DePuy) | NR | NA | a, b, c, d, e | n, q, r | 70±6.3 | 41/19 | 27.7±4.0 |
|  |  |  |  | CON | 60 |  |  |  |  |  |  | 70±6.3 | 41/19 | 27.7±4.0 |
| 18 | Kim et al. | 2009 | Korea | CAS | 160 | Image-free navigation system (BrainLab, Depuy) | NexGen CR-Flex (Zimmer) | Yes | 3.4 years | a, b, c, d, e | - | 68.5 | 141/19 | 26.8 |
|  |  |  |  | CON | 160 |  |  |  |  |  |  | 68.5 | 141/19 | 26.8 |
| 19 | Choong et al. | 2009 | Australia | CAS | 57 | Image-free navigation system (BrainLab, Depuy) | PFC Sigma (DePuy) | Yes | 6 weeks | a | n | 70 (median) | 40/17 | 29.5 (median) |
|  |  |  |  | CON | 54 |  |  |  |  |  |  | 69 (median) | 27/27 | 29.5 (median) |
| 20 | Seon and Song | 2009 | Korea | CAS | 43 | Image-free knee navigation system (OrthoPilot, Aesculap) | NR | No | 2 years | a, b, c | o | 67.2 | 41/2 | NR |
|  |  |  |  | CON | 42 |  |  |  |  |  |  | 67.6 | 38/4 |  |
| 21 | Lee et al. | 2010 | Korea | CAS | 60 | Image-free knee navigation system (OrthoPilot, Aesculap) | E-motion prosthesis (Aesculap) | Yes | 2.3 years | a | o | 66 | 57/3 | NR |
|  |  |  |  | CON | 56 |  |  |  |  |  |  | 67 | 54/2 |  |
| 22 | Zhang et al. | 2011 | China | CAS | 32 | Image-free navigation system (BrainLab, Depuy) | Gemini MK II knee implants (Link) | NR | 6 months | b, c | q | NR | NR | NR |
|  |  |  |  | CON | 32 |  |  |  |  |  |  |  |  |  |
| 23 | Barrett et al. | 2011 | USA | CAS | 81 | Image-free navigation system (BrainLab, Depuy) | PFC Sigma (DePuy) | NR | 1 year | - | f, g, h, q | 66.3±8.0 | 36/45 | 31.7±5.6 |
|  |  |  |  | CON | 85 |  |  |  |  |  |  | 64.5±7.6 | 56/29 | 31.7±6.1 |
| 24 | Pang et al. | 2011 | Singapore | CAS | 70 | Image-free navigation system (BrainLab, Depuy) | PFC Sigma (DePuy) | No | 2 years | a | f, g, i, j, k, m, n, o | 68 | 60/10 | 29.3±4.3 |
|  |  |  |  | CON | 70 |  |  |  |  |  |  | 70 | 58/12 | 28.5±5.1 |
| 25 | Hiscox et al. | 2011 | Canada | CAS | 61 | Image-free knee navigation system (Stryker) | Duracon condylar TKA (Stryker) | NR | 1.2 years | a | n | 66.8±10.3 | 37/24 | NR |
|  |  |  |  | CON | 59 |  |  |  |  |  |  | 67.5±9.7 | 42/17 |  |
| 26 | Harvie et al. | 2012 | Australia | CAS | 24 | Image-free knee navigation system (Stryker) | Duracon condylar TKA (Stryker) | No | 5 years | - | h, l | 70 | 15/9 | NR |
|  |  |  |  | CON | 22 |  |  |  |  |  |  | 70.1 | 13/9 |  |
| 27 | Kim et al. | 2012 | Korea | CAS | 520 | Image-free navigation system (BrainLab, Depuy) | PFC Sigma (DePuy)/NexGen LPS-Flex (Zimmer) | Yes | 10.8 years | a, b, c, d, e | - | 68 | 452/68 | 27.8 |
|  |  |  |  | CON | 520 |  |  |  |  |  |  | 68 | 452/68 | 27.8 |
| 28 | Hoffart et al. | 2012 | Germany | CAS | 98 | Image-free knee navigation system (PiGalileo) | TC-Plus Solution (Smith & Nephew) | Yes | 5 years | a | n, q, r | 70.9±10.8 | 71/27 | 29.1±6.5 |
|  |  |  |  | CON | 97 |  |  |  |  |  |  | 69.2±11.8 | 68/29 | 30.3±5.8 |
| 29 | Zhang et al. | 2012 | China | CAS | 41 | Image-free knee navigation system (Stryker) | Scorpio PS System (Stryker) | No | 6 months | a | o | 66.3 | 25/16 | NR |
|  |  |  |  | CON | 41 |  |  |  |  |  |  | 63.3 | 29/12 |  |
| 30 | Lützner et al. | 2013 | Germany | CAS | 34 | Image-free knee navigation system (Stryker) | Scorpio PS System (Stryker) | No | 5 years | a | j, k | 68.1±9.0 | 25/9 | 31.1±5.3 |
|  |  |  |  | CON | 33 |  |  |  |  |  |  | 66.9±9.8 | 20/13 | 31.0±4.4 |
| 31 | Cip et al. | 2014 | Austria | CAS | 73 | Image-free navigation system (BrainLab, Depuy) | NexGen LPS-Flex (Zimmer) | NR | 5 years | a, b, c, e | j, k, l, o | 74.9±8.6 | 67/24 | 30.2±5.4 |
|  |  |  |  | CON | 75 |  |  |  |  |  |  | 76.1±7.0 | 64/28 | 28.2±4.7 |
| 32 | Gøthesen et al. | 2014 | Norway | CAS | 95 | Image-free navigation system (BrainLab, Depuy) | CR Profix total knee prosthesis (Smith & Nephew) | No | 1 year | a, b, c, d, e | n, q | 68.3±7.8 | 58/37 | NR |
|  |  |  |  | CON | 94 |  |  |  |  |  |  | 67.7±6.8 | 59/35 |  |
| 33 | Blyth et al. | 2015 | UK | CAS | 88 | Image-based navigation system (iNav, Medtronic) | NexGen LPS-Flex (Zimmer) | NR | 1 year | a | n | 65.6±10.5 | 56/45 | NR |
|  |  |  |  | CON | 81 |  |  |  |  |  |  | 65.4±10.8 | 60/37 |  |
| 34 | Maderbacher  et al. | 2015 | Germany | CAS | 40 | Image-free navigation system (BrainLab, Depuy) | PFC Sigma (DePuy) | NR | NA | a, b, c | q | 69.8±8.7 | 28/12 | 31.2±5.3 |
|  |  |  |  | CON | 40 |  |  |  |  |  |  | 69.5±8.9 | 25/15 | 32.0±5.0 |
| 35 | Chen et al. | 2015 | Singapore | CAS | 50 | Image-free navigation system (BrainLab, Depuy) | PFC Sigma (DePuy) | Yes | NA | a, b | - | 67±9 | 32/18 | 27±3 |
|  |  |  |  | CON | 50 |  |  |  |  |  |  | 67±8 | 35/15 | 29±6 |
| 36 | Zhang et al. | 2016 | China | CAS | 18 | Image-based navigation system (VSG) | NR | NR | 1 year | - | q, r | 63.3±5.1 | 7/11 | NR |
|  |  |  |  | CON | 18 |  |  |  |  |  |  | 62.1±4.9 | 5/13 |  |
| 37 | Liu and Liu | 2016 | China | CAS | 38 | Image-based navigation system | Johnson & Johnson | NR | 1 year | - | q | 57.12±10.25 | 22/16 | NR |
|  |  |  |  | CON | 41 |  |  |  |  |  |  | 56.10±11.15 | 23/18 |  |
| 38 | Thiengwittayaporn  et al. | 2016 | Thailand | CAS | 40 | Handheld navigation system (iAssist, Zimmer) | Legion PS system (Smith & Nephew) | NR | 6 weeks | a, b, c, d, e | q, r | 68.0±8.0 | 32/8 | 26.6±3.7 |
|  |  |  |  | CON | 40 |  |  |  |  |  |  | 65.9±6.3 | 34/6 | 26.2±3.2 |
| 39 | Zhu et al. | 2016 | China | CAS | 30 | Image-free navigation system (BrainLab, Depuy) | PFC Sigma (DePuy) | Yes | 9.1 years | - | o | 67.9±8.1 | 28/2 | 27.6±4.7 |
|  |  |  |  | CON | 37 |  |  |  |  |  |  | 65.3±7.4 | 31/6 | 27.7±4.5 |
| 40 | Song et al. | 2016 | Korea | CAS | 39 | Image-free knee navigation system (OrthoPilot, Aesculap) | E-motion prosthesis (Aesculap) | NR | 10 years | a | j, k, l, o | 65.4±5.9 | 29/10 | NR |
|  |  |  |  | CON | 41 |  |  |  |  |  |  | 66.1±8.1 | 31/10 |  |
| 41 | Gharaibeh et al. | 2017 | Australia | CAS | 89 | Handheld navigation system (OrthAlign) | Legion PS system (Smith & Nephew) | Yes | NA | a, b, c, d, e | q | 69.2 ± 8.7 | 55/34 | 29.6 ± 5.4 |
|  |  |  |  | CON | 90 |  |  |  |  |  |  | 69 ± 8.3 | 50/39 | 29.2 ± 4.8 |
| 42 | Kim et al. | 2017 | Korea | CAS | 162 | Image-free navigation system (BrainLab, Depuy) | NexGen CR-Flex (Zimmer) | NR | 12.3 years | a, b, c, d, e | o | 68.1±7.5 | 153/9 | 27 ± 3.3 |
|  |  |  |  | CON | 162 |  |  |  |  |  |  | 68.1±7.5 | 153/9 | 27 ± 3.3 |
| 43 | Todesca et al. | 2017 | Italy | CAS | 121 | Image-based navigation system (Amplivision) | Amplitude | NR | 6.4 years | a, b, c, d, e | - | NR | NR | NR |
|  |  |  |  | CON | 117 |  |  |  |  |  |  |  |  |  |
| 44 | Kim et al. | 2018 | Korea | CAS | 282 | Image-free navigation system (BrainLab, Depuy) | NexGen LPS-Flex (Zimmer) | NR | 15 years | a, b, c | j, k, l, o | 59±7 | 223/59 | 28±8 |
|  |  |  |  | CON | 282 |  |  |  |  |  |  | 59±7 | 223/59 | 28±8 |
| 45 | Kinney et al. | 2018 | USA | CAS | 25 | Handheld navigation system (iAssist, Zimmer) | Persona PS components (Zimmer) | NR | NA | b, c | n | 66.4±2.3 | 13/12 | 20.4±1.2 |
|  |  |  |  | CON | 25 |  |  |  |  |  |  | 65.0±2.0 | 16/9 | 31.1±1.2 |
| 46 | Petursson et al. | 2018 | Norway | CAS | 87 | Image-free navigation system (BrainLab, Depuy) | CR Profix total knee prosthesis (Smith & Nephew) | NR | 2 years | - | j, k | 67.9±6.8 | 51/36 | 27.7±3.5 |
|  |  |  |  | CON | 80 |  |  |  |  |  |  | 67.6±6.6 | 51/29 | 28.5±3.8 |
| 47 | Zhu et al. | 2018 | Singapore | CAS | 46 | Image-free navigation system (BrainLab, Depuy) | PFC Sigma (DePuy) | NR | 2 years | - | o | 66.4±9.3 | 29/17 | 27.1±3.0 |
|  |  |  |  | CON | 48 |  |  |  |  |  |  | 66.9±7.9 | 34/14 | 29.1±6.0 |
| 48 | Xu et al. | 2019 | China | CAS | 39 | Handheld navigation system (i-JOIN) | Genesis II PS (Smith & Nephew) | NR | 1 week | a | n, q, r | 65.28±6.77 | 30/9 | NR |
|  |  |  |  | CON | 40 |  |  |  |  |  |  | 65.33±7.59 | 31/9 |  |
| 49 | Hsu et al. | 2019 | China | CAS | 56 | Image-free navigation system (BrainLab, Depuy) | PFC Sigma (DePuy) | No | 8.1 years | a, b, c, d, e | l, n, o, q, r | 68.7±5.8 | 44/12 | 28.8±4.1 |
|  |  |  |  | CON | 56 |  |  |  |  |  |  | 68.7±5.8 | 44/12 | 28.8±4.1 |
| 50 | d'Amato et al. | 2019 | Italy | CAS | 48 | Image-free knee navigation system (Stryker) | Scorpio Posterior Stabilized System (Stryker)/Optetrak Posterior Stabilized System (Exatech) | NR | 10.3 years | - | j, k | 68.8±6.3 | 30/30 | NR |
|  |  |  |  | CON | 45 |  |  |  |  |  |  | 71.1±7.8 | 30/30 |  |
| 51 | Selvanayagam  et al. | 2019 | India | CAS | 25 | Image-free knee navigation system (OrthoPilot, Aesculap) | Columbus CR (Aesculap) | No | 4.6 years | a, b | f, g, h, j, k, l | 63.2 | NR | 28.7 |
|  |  |  |  | CON | 25 |  |  |  |  |  |  | 62.7 |  | 28.7 |
| 52 | Tsuda et al. | 2021 | Japan | CAS | 42 | Handheld navigation system (iAssist, Zimmer) | Persona PS or CR (Biomet) | NR | 6 months | b, c, d, e | g, I, o, q, r | 74.2±8.1 | 35/7 | 25.4±4.0 |
|  |  |  |  | CON | 41 |  |  |  |  |  |  | 75.5±9.0 | 31/10 | 26.2±4.4 |
| 53 | Zheng et al. | 2021 | China | CAS | 21 | Image-free knee navigation system (OrthoPilot, Aesculap) | E-motion prosthesis (Aesculap) | NR | 6 months | a, b, c, d, e | h, n, o, q, r | 66.95±6.68 | 19/2 | 26.74±3.21 |
|  |  |  |  | CON | 21 |  |  |  |  |  |  | 66.95±6.68 | 19/2 | 26.74±3.21 |
| 54 | Ali et al. | 2021 | Australia | CAS | 81 | Handheld navigation system (OrthAlign) | Legion PS system (Smith & Nephew) | Yes | 4.3 years | - | l, n | 68.1 | 57/32 | NR |
|  |  |  |  | CON | 78 |  |  |  |  |  |  | 68.2 | 50/39 |  |
| 55 | Narkbunnam  et al. | 2022 | Thailand | CAS | 30 | Handheld navigation system (iAssist, Zimmer) | NexGen LPS-Flex (Zimmer) | No | 6 weeks | a, b, c, d, e | f, g, j, k, o, q | 67.5±7.6 | 27/3 | 28.2±5.8 |
|  |  |  |  | CON | 30 |  |  |  |  |  |  | 66.4±6.3 | 27/3 | 27.3±4.4 |
| 56 | Jagadeesh et al. | 2022 | India | CAS | 35 | NR | NR | No | 1.9 years | b, c | f, g, i, j, k, m | 61.88 | 23/12 | NR |
|  |  |  |  | CON | 35 |  |  |  |  |  |  | 63.85 | 23/12 |  |
| 57 | Farhan-Alanie  et al. | 2022 | UK | CAS | 101 | Image-based navigation system (iNav, Zimmer) | NexGen LPS-Flex (Zimmer) | NR | 10 years | a | f, g, i, j, k, m, n, o | 65.3±10.0 | 55/46 | NR |
|  |  |  |  | CON | 98 |  |  |  |  |  |  | 69.4±9.3 | 60/38 |  |
| 58 | Yen et al. | 2023 | China | CAS | 50 | Image-free knee navigation system (Stryker) | NexGen LPS-Flex (Zimmer) | NR | 3 months | a, b, c | n, r | 68.78±6.20 | 39/11 | 27.00±4.08 |
|  |  |  |  | CON | 50 |  |  |  |  |  |  | 70.54±5.80 | 40/10 | 27.87±4.15 |
| 59 | Jarusriwanna et al. | 2023 | Thailand | CAS | 31 | Handheld navigation system (iAssist, Zimmer) | NexGen LPS-Flex (Zimmer) | NR | 5 days | - | n, q, r | 70.9±6.0 | 27/4 | 26.7±3.3 |
|  |  |  |  | CON | 32 |  |  |  |  |  |  | 70.0±6.6 | 30/3 | 28.1±4.1 |
| 60 | Pietsch et al. | 2013 | Austria | PSI | 40 | PSI (Zimmer) | NexGen LPS-Flex (Zimmer) | NR | 3 months | - | f, g, o, p, q, r | 71.4±6.6 | 27/13 | 29.0±3.5 |
|  |  |  |  | CON | 40 |  |  |  |  |  |  | 69.2±9.4 | 21/19 | 30.8±4.9 |
| 61 | Boonen et al. | 2013 | Netherlands | PSI | 86 | Materialise (Leuven) | Vanguard Complete Knee System (Biomet) | NR | 6 weeks | a, b, c, d, e | q | 69±8.0 | 56/34 | 30.3 |
|  |  |  |  | CON | 82 |  |  |  |  |  |  | 65±8.8 | 50/40 | 29.5 |
| 62 | Hamilton et al. | 2013 | Virginia | PSI | 26 | TruMatch (DePuy) | PFC PS total knee prosthesis (DePuy) | NR | NA | a, b, c, d, e | - | 68.1 | 12/14 | 30.9 |
|  |  |  |  | CON | 26 |  |  |  |  |  |  | 67.6 | 19/7 | 31.1 |
| 63 | Chareancholvanich  et al. | 2013 | Thailand | PSI | 40 | PSI (Zimmer) | NexGen LPS-Flex (Zimmer) | No | 4 weeks | a, b, c | q, r | 69.5±7.3 | 34/6 | 27.7±6.0 |
|  |  |  |  | CON | 40 |  |  |  |  |  |  | 70.3±8.0 | 36/4 | 28±4.4 |
| 64 | Chotanaphuti  et al. | 2014 | Thailand | PSI | 40 | TruMatch (DePuy) | PFC Sigma (DePuy) | Yes | 6 weeks | a | q, r | 69.7±5.5 | NR | 25.0±2.4 |
|  |  |  |  | CON | 40 |  |  |  |  |  |  | 69.3±5.5 |  | 25.0±2.1 |
| 65 | Woolson et al. | 2014 | USA | PSI | 22 | TruMatch (DePuy) | NR | Yes | 6 months | a, b, c, d, e | - | NR | NR | NR |
|  |  |  |  | CON | 26 |  |  |  |  |  |  |  |  |  |
| 66 | Abdel et al. | 2014 | USA | PSI | 20 | NR | Negev LPS-Flex mobile (Zimmer) | Yes | 3 months | - | f, n | 71±5 | 12/8 | 28.3±3.9 |
|  |  |  |  | CON | 20 |  |  |  |  |  |  | 71±7 | 12/8 | 30±4 |
| 67 | Kotela and Kotela | 2014 | Poland | PSI | 49 | Signature (Biomet) | Vanguard Complete Knee System (Biomet) | No | 1 year | a, b, c, d, e | f, g, h | 66.1±8.4 | 33/16 | 30.0±4.6 |
|  |  |  |  | CON | 46 |  |  |  |  |  |  | 68.6±9.9 | 33/13 | 29.6±5.6 |
| 68 | Gan et al. | 2015 | China | PSI | 35 | Materialise (Leuven) | Scorpio PS System (Stryker) | NR | NA | a, b, c | q, r | 68.5±4.8 | 25/10 | NR |
|  |  |  |  | CON | 35 |  |  |  |  |  |  | 67.8±3.4 | 26/9 |  |
| 69 | Abane et al. | 2015 | France | PSI | 59 | Visionaire system (Smith & Nephew) | Genesis II PS (Smith & Nephew) | Yes | 3 months | a, b, c, d, e | f, g, I, q, r | 67.8±9.3 | 47/23 | 28.8±5 |
|  |  |  |  | CON | 67 |  |  |  |  |  |  | 70.4±7.3 | 41/29 | 28.6±5 |
| 70 | Molicnik et al. | 2015 | Slovenia | PSI | 19 | Signature (Biomet) | Vanguard Complete Knee System (Biomet) | NR | NA | a | - | 67.1±7.1 | 17/2 | 31.9±5.3 |
|  |  |  |  | CON | 19 |  |  |  |  |  |  | 66.8±6.7 | 14/5 | 33.3±5.5 |
| 71 | Kotela et al. | 2015 | Poland | PSI | 49 | Signature (Biomet) | Vanguard Complete Knee System (Biomet) | No | 1 year | - | p | 66.1±8.4 | 33/16 | 30.0±4.6 |
|  |  |  |  | CON | 46 |  |  |  |  |  |  | 68.6±9.9 | 33/13 | 29.6±5.6 |
| 72 | Huijbregts et al. | 2016 | Australia | PSI | 69 | Visionaire (Smith & Nephew) | Genesis II/Legion systems (Smith & Nephew) | NR | 6 weeks | a, b, c, d, e | n, q | 66.7±9.1 | 40/29 | NR |
|  |  |  |  | CON | 64 |  |  |  |  |  |  | 69.0±9.6 | 32/32 |  |
| 73 | Boonen et al. | 2016 | Netherlands | PSI | 82 | Materialise (Leuven) | Vanguard Complete Knee System (Biomet) | NR | 3.7 years | - | f, h, i, j, l, m, n, p | 69±8.0 | 56/34 | 30.3 |
|  |  |  |  | CON | 81 |  |  |  |  |  |  | 65±8.8 | 50/40 | 29.5 |
| 74 | Anderl et al. | 2016 | Austria | PSI | 114 | MyKnee (Medacta) | GMK Primary (Medacta) | No | 2 years | a, b, c, d, e | j, k, o, p | 68.7±8.2 | 60/54 | 29.9±5.2 |
|  |  |  |  | CON | 108 |  |  |  |  |  |  | 67.7±9.6 | 63/45 | 29.8±5.4 |
| 75 | Qiu et al. | 2017 | China | PSI | 10 | Arigin 3D Surgical Templating | NR | NR | NA | a, b, c | - | 67.57±7 | 6/4 | NR |
|  |  |  |  | CON | 16 |  |  |  |  |  |  | 65.5±6.58 | 14/2 |  |
| 76 | Vide et al. | 2017 | Portugal | PSI | 47 | Visionaire (Smith & Nephew) | TC Plus Total Knee System (Smith & Nephew) | No | NA | a | q | 67.8±8.4 | 32/15 | 31 |
|  |  |  |  | CON | 48 |  |  |  |  |  |  | 69.3±6.5 | 33/15 | 30.3 |
| 77 | Tammachote  et al. | 2018 | Thailand | PSI | 51 | Visionaire (Smith & Nephew) | Genesis II PS (Smith & Nephew) | No | 2 years | a, b, c | h, l, o, q, r | 72±7 | 42/12 | 25±4 |
|  |  |  |  | CON | 51 |  |  |  |  |  |  | 72±8 | 39/15 | 26±5 |
| 78 | Stolarczyk et al. | 2018 | Poland | PSI | 30 | Visionaire (Smith & Nephew) | NR | NR | 3 months | - | f, g, p, q | 70.2±5.9 | 22/8 | 30.4±4.4 |
|  |  |  |  | CON | 30 |  |  |  |  |  |  | 69.6±7.1 | 18/12 | 31.6±5.4 |
| 79 | Maus et al. | 2018 | Germany | PSI | 59 | Imprint (Aesculap) | Aesculap AG | NR | 3 months | a | f, g, o, q, r | 68.1±8.5 | 33/26 | 31.8±6.1 |
|  |  |  |  | CON | 66 |  |  |  |  |  |  | 71.5±8.1 | 43/23 | 30.6±5.3 |
| 80 | Van Leeuwen  et al. | 2018 | Norway | PSI | 44 | Materialise (Leuven) | Vanguard Complete Knee System (Biomet) | NR | 2 years | a, b, c, d, e | q | 67 | 30/14 | 31 |
|  |  |  |  | CON | 50 |  |  |  |  |  |  | 64 | 32/18 | 29 |
| 81 | Kosse et al. | 2018 | Netherlands | PSI | 21 | Visionaire (Smith & Nephew) | Genesis II PS (Smith & Nephew) | Yes | 1 year | - | q | 62.7±4.5 | 13/8 | 28.1±3.3 |
|  |  |  |  | CON | 21 |  |  |  |  |  |  | 63.4±4.2 | 12/9 | 27.8±3.1 |
| 82 | Sariali et al. | 2019 | France | PSI | 40 | BoneSurfacer (Symbios) | NR | NR | 3 months | a, b, c | o, q | 67.7±8.7 | 33/7 | 29.7±5.3 |
|  |  |  |  | CON | 40 |  |  |  |  |  |  | 68.9±9.2 | 24/16 | 29.5±4.5 |
| 83 | Turgeon et al. | 2019 | Canada | PSI | 25 | Visionaire (Smith & Nephew) | Legion PS system (Smith & Nephew) | NR | 2 years | a | n, q | 63.8±9.2 | 18/7 | 33.6±7.2 |
|  |  |  |  | CON | 29 |  |  |  |  |  |  | 65.7±9.2 | 22/7 | 32.0±6.0 |
| 84 | Schotanus  et al. | 2019 | Netherlands | PSI | 83 | PSI (Zimmer) | Vanguard Complete Knee System (Biomet) | NR | 5.1 years | - | f, h, i, j, l, m, p | NR | NR | NR |
|  |  |  |  | CON | 80 |  |  |  |  |  |  |  |  |  |
| 85 | Cucchi et al. | 2019 | Germany | PSI | 12 | TruMatch (DePuy) | PFC Sigma (DePuy) | Yes | 2 months | - | p, q | 74.25 | 9/3 | 28.7 |
|  |  |  |  | CON | 10 |  |  |  |  |  |  | 69.2 | 8/2 | 27.9 |
| 86 | Sun et al. | 2020 | China | PSI | 40 | Materialise (Leuven) | NR | No | 9 months | - | f, o, q, r | 68.7±9.1 | 32/8 | 25.8±3.9 |
|  |  |  |  | CON | 40 |  |  |  |  |  |  | 67.6±8.4 | 33/7 | 26.6±2.2 |
| 87 | Li et al. | 2020 | China | PSI | 58 | Materialise (Leuven) | EVOLUTION medial Priot | No | 7.4 months | - | f, p, q, r | 68.28±8.56 | 50/8 | NR |
|  |  |  |  | CON | 52 |  |  |  |  |  |  | 69.55±7.43 | 46/6 |  |
| 88 | Mehdipour  et al. | 2020 | Iran | PSI | 12 | NR | Deep Dish prostheses (Corin) | No | 2 years | a, b, c | l, q | 60.3±10.4 | 6/6 | 29.7±1.8 |
|  |  |  |  | CON | 12 |  |  |  |  |  |  | 62.6±8.7 | 8/4 | 29.2±1.1 |
| 89 | Sun et al. | 2020 | China | PSI | 30 | Siemens | NR | No | 11 months | - | f | 65.2 | 22/8 | NR |
|  |  |  |  | CON | 30 |  |  |  |  |  |  | 62.7 | 24/6 |  |
| 90 | Huang et al. | 2021 | China | PSI | 20 | Siemens | MicroPort EVOLUTION | NA | 3 months | a | f, p, q | 69.6±7.8 | 7/13 | 23.28±4.21 |
|  |  |  |  | CON | 19 |  |  |  |  |  |  | 71.8±8.1 | 9/10 | 22.28±4.39 |
| 91 | Sood and Yasin | 2021 | India | PSI | 50 | NR | NRG (*Stryker*) | NR | 1 year | a | h, n, q | 64.08±7.36 | 28/22 | 26.69±2.15 |
|  |  |  |  | CON | 50 |  |  |  |  |  |  | 62.76±7.14 | 26/24 | 26.78±1.63 |
| 92 | Li et al. | 2021 | China | PSI | 38 | Materialise (Leuven) | NR | No | 1 year | - | h | 69.0±6.2 | 32/6 | 27.8±3.5 |
|  |  |  |  | CON | 36 |  |  |  |  |  |  | 69.1±6.2 | 27/9 | 28.4±3.7 |
| 93 | Hampton et al. | 2022 | UK | PSI | 38 | PSI (Zimmer) | NexGen CR and NexGen LPS-flex (Zimmer) | NR | 5 years | a | i, m, n, q, r | 71±3 | 28/17 | 30.3±3.0 |
|  |  |  |  | CON | 39 |  |  |  |  |  |  | 68±3 | 30/15 | 30.9±3.8 |
| 94 | Rivrud et al. | 2023 | Netherlands | PSI  CON | 37 | Signature (Biomet) | Vanguard Complete Knee System (Biomet) | NR | 5 years | - | o, p | 67±8 | 24/13 | 30.0±4 |
|  |  |  |  |  | 40 |  |  |  |  |  |  | 63±7 | 27/13 | 29.6±5 |
| 95 | Sadoghi et al. | 2023 | Austria | PSI  CON | 50 | MyKnee (Medacta) | GMK Sphere (Medacta) | NR | 2 weeks | e | - | 69±8.8 | 34/16 | 30±6.1 |
|  |  |  |  |  | 50 |  |  |  |  |  |  | 71±8.3 | 31/19 | 30±4.8 |
| 96 | Song et al. | 2011 | Korea | RAS | 30 | ROBODOC | NR | NR | 1.3 years | a, b, c, d, e | h, o, q, r | 67±6.3 | 30/0 | 27±6.5 |
|  |  |  |  | CON | 30 |  |  |  |  |  |  | 67±6.3 | 30/0 | 27±6.5 |
| 97 | Song et al. | 2013 | Korea | RAS | 50 | ROBODOC | NexGen LPS-Flex (Zimmer) | NR | 5.4 years | a, b, c, d, e | l, o, q, r | 66.1±7.1 | 46/4 | 26.3±2.7 |
|  |  |  |  | CON | 50 |  |  |  |  |  |  | 64.8±5.3 | 45/5 | 26.2±3.9 |
| 98 | Liow et al. | 2014 | Singapore | RAS | 31 | ORTHODOC | NexGen LPS-Flex (Zimmer) | NR | 6 months | a | f, g, I, o, q | 67.5±8.6 | NR | 27.5±3.8 |
|  |  |  |  | CON | 29 |  |  |  |  |  |  | 68.3±7.7 |  | 27.2±4.9 |
| 99 | Liow et al. | 2017 | Singapore | RAS | 31 | ORTHODOC | NexGen LPS-Flex (Zimmer) | NR | 2 years | - | i, j, k, n | 67.5±8.6 | NR | 27.5±3.8 |
|  |  |  |  | CON | 29 |  |  |  |  |  |  | 68.3±7.7 |  | 27.2±4.9 |
| 100 | Cho et al. | 2019 | Korea | RAS | 160 | ROBODOC | NexGen LPS-Flex (Zimmer) | NR | 10 years | a, b, c, d, e | k, l, o | 68.2±5.8 | 141/14 | NR |
|  |  |  |  | CON | 230 |  |  |  |  |  |  | 67.6±6.3 | 163/33 |  |
| 101 | Kim et al. | 2020 | Korea | RAS | 724 | ROBODOC | Duracon condylar TKA (Stryker) | NR | 13 years | a, b, c, d, e | j, l, n | 60±7 | 542/132 | 28±9 |
|  |  |  |  | CON | 724 |  |  |  |  |  |  | 61±8 | 530/144 | 29±8 |
| 102 | Sun et al. | 2021 | China | RAS | 30 | SkyWalker | NR | No | 3 months | - | f, g | 67.1 | 24/6 | 25.3±3.4 |
|  |  |  |  | CON | 30 |  |  |  |  |  |  | 65.9 | 25/5 | 25.6±2.8 |
| 103 | Thiengwittayaporn  et al. | 2021 | Thailand | RAS | 75 | NAVIO | Legion PS system (Smith & Nephew) | NR | 6 weeks | a, b, c, d, e | - | 69.0±8.3 | 40/35 | 28.0±4.9 |
|  |  |  |  | CON | 77 |  |  |  |  |  |  | 69.1±7.3 | 45/32 | 27.7±4.6 |
| 104 | Yuan et al. | 2021 | China | RAS | 28 | YUANHUA-TKA | NR | NR | 3 months | - | f, o, p, q, r | 65.2 | 19/9 | 27.4±3.0 |
|  |  |  |  | CON | 32 |  |  |  |  |  |  | 65.4 | 28/4 | 28.4±3.6 |
| 105 | Xu et al. | 2022 | China | RAS | 37 | YUANHUA-TKA | Unique knee (Zhengtian) | Yes | 3 months | a, b, c, d, e | h, q | 64.5±5.3 | 26/11 | 26.2±3.8 |
|  |  |  |  | CON | 35 |  |  |  |  |  |  | 63.4±7.2 | 28/7 | 26.4±3.1 |
| 106 | Li et al. | 2022 | China | RAS | 73 | HURWA | Legion PS system (Smith & Nephew) | NR | 3 months | a | h, o | 68.0±7.97 | 60/13 | 26.9±3.26 |
|  |  |  |  | CON | 77 |  |  |  |  |  |  | 69.0±6.00 | 62/15 | 27.4±3.38 |
| 107 | Bollars et al. | 2023 | Belgium | RAS | 26 | NAVIO (Smith & Nephew) | Journey Ⅱ (Smith & Nephew) | yes | NR | a, b, c, d | - | 64.4±8.7 | 15/11 | 28.7±4.4 |
|  |  |  |  | CON | 26 |  |  |  |  |  |  | 66.4±7.2 | 17/9 | 28.7±4.7 |
| 108 | Li et al. | 2023 | China | RAS | 66 | NR | NR | NR | 3 months | - | j, q, r | 64.5±6.3 | 57/9 | 25.9±3.0 |
|  |  |  |  | CON | 61 |  |  |  |  |  |  | 65.0±5.5 | 45/16 | 26.0±2.6 |
| 109 | Clement et al. | 2023 | UK | RAS | 50 | MAKO (Stryker) | NR | NR | 6 months | - | j, l | 66.8±8.7 | 24/26 | 31.7±5.5 |
|  |  |  |  | CON | 50 |  |  |  |  |  |  | 66.7±9.6 | 21/29 | 31.3±6.8 |
| 110 | Tian et al. | 2023 | China | RAS | 62 | Jianjia Robot | Persona PS or CR (Biomet) | NR | 3 months | a, b, c, d, e | f, n, o, p, q | 68.17±7.59 | 49/13 | 25.62±3.08 |
|  |  |  |  | CON | 61 |  |  |  |  |  |  | 68.84±7.12 | 46/15 | 26.64±3.33 |
| 111 | Wang et al. | 2021 | China | CAS | 40 | Image-free knee navigation system (OrthoPilot, Aesculap) | NR | NR | 3 months | a, b, c, d, e | q | 68±6 | 34/6 | 27.6±3.7 |
|  |  |  |  | PSI | 40 | Materialise (Leuven) |  |  |  |  |  | 69±6 | 35/5 | 27.6±3.1 |
| 112 | Yan et al. | 2015 | China | CAS | 30 | Image-free navigation system (BrainLab, Depuy) | NexGen LPS-Flex (Zimmer) | NR | 3 months | a, b, c, d, e | f, g, I, o, q | 66.7±7.2 | 26/4 | NR |
|  |  |  |  | PSI | 30 | Materialise (Leuven) |  |  |  |  |  | 67.5±8 | 17/13 |  |
|  |  |  |  | CON | 30 |  |  |  |  |  |  | 69.5±8.4 | 24/6 |  |

Note: CAS = computer-assisted navigation systems; CON = conventional cutting instruments; PSI = patient-specific instruments; RAS = robot-assisted systems; NR = not report.

Note for outcomes: a mechanical axis outliers; b coronal femoral component angle outliers; c coronal tibial component angle outliers; d sagittal femoral component angle outliers; e sagittal tibial component angle outliers; f short-term Knee Society Score (KSS) knee scores; g medium-and-long-term KSS knee scores; h short-term KSS function scores; i medium-and-long-term KSS function scores; j short-term Western Ontario and McMaster Universities Osteoarthritis Index (WOMAC) scores; k medium-and-long-term WOMAC scores; l short-term Oxford knee score (OKS); m medium-and-long-term OKS; n postoperative complications; o range of motion; p visual analogue scale scores; q operative time; r total blood loss.

Fig. 4 History of assistive techniques appearance and frequency. *Y-axis* represents the number of groups per year.

**Supplement 3:** **Heterogeneity of the direct comparisons**

Table 2. Heterogeneity of the direct comparisons (*I^2^*)

| Outcome | CON vs CAS | CON vs PSI | CON vs RAS | CAS vs PSI |
| --- | --- | --- | --- | --- |
| Mechanical axis outliers | 29.4% | 29.1% | 48.5% | 0.0% |
| Coronal femoral component angle outliers | 42.8% | 49.6% | 34.3% | 0.0% |
| Coronal tibial component angle outliers | 43.7% | **55.5%** | **56.7%** | 0.0% |
| Sagittal femoral component angle outliers | 40.5% | 25.6% | 42.5% | 0.0% |
| Sagittal tibial component angle outliers | **82.4%** | **52.9%** | **55.7%** | 0.0% |
| Short-term KSKS | 30.0% | **60.1%** | 0.0% | - |
| Short-term KSFS | **80.8%** | 30.4% | 0.0% | - |
| Short-term WOMAC scores | 0.0% | 0.0% | 0.6% | - |
| Short-term OKS scores | **88.6%** | 0.0% | 0.0% | - |
| Medium-and-long-term KSKS | **62.1%** | 0.0% | **77.9%** | - |
| Medium-and-long-term KSFS | **66.6%** | - | 0.0% | - |
| Medium-and-long-term WOMAC scores | 49.2% | **72.4%** | 0.0% | - |
| Medium-and-long-term OKS scores | **87.0%** | 37.9% |  | - |
| Postoperative complications | 0.0% | 31.5% | 0.0% | - |
| ROM | 40.1% | 0.0% | 0.0% | - |
| VAS scores | 0.0% | **65.8%** | 0.0% | - |
| Operative time | **95.0%** | **98.7%** | **91.8%** | **77.4%** |
| Total blood loss | **69.6%** | **94.7%** | **87.8%** | - |

CON = conventional cutting instruments; CAS = computer-assisted navigation systems; PSI = patient-specific instruments; RAS = robot-assisted systems; KSKS = Knee Society Knee Score; KSFS = Knee Society Function Score; WOMAC = Western Ontario and McMaster Universities Osteoarthritis Index; OKS = Oxford Knee Score; ROM = range of motion; VAS = visual analog scale.

**Supplement 4:** **Surface under the cumulative ranking score of all outcomes**

Table 3. Surface under the cumulative ranking score of radiological outcomes

|  | Mechanical axis | Coronal femoral component angle | Coronal tibial component angle | Sagittal femoral component angle | Sagittal tibial component angle |
| --- | --- | --- | --- | --- | --- |
| CON | 0.01 | 0.004 | 0.165 | 0.172 | 0.276 |
| CAS | 0.712 | 0.644 | 0.867 | 0.686 | 0.68 |
| PSI | 0.324 | 0.431 | 0.208 | 0.166 | 0.058 |
| RAS | 0.954 | 0.92 | 0.759 | 0.976 | 0.986 |

Table 4. Surface under the cumulative ranking score of clinical outcome scores

|  | Short-term KSKS | Short-term KSFS | Short-term WOMAC scores | Short-term OKS scores | Medium-and-long-term KSKS | Medium-and-long-term KSFS | Medium-and-long-term WOMAC scores | Medium-and-long-term OKS scores |
| --- | --- | --- | --- | --- | --- | --- | --- | --- |
| CON | 0.401 | 0.404 | 0.46 | 0.347 | 0.276 | 0.209 | 0.223 | 0.529 |
| CAS | 0.294 | 0.84 | 0.787 | 0.379 | 0.695 | 0.549 | 0.236 | 0.558 |
| PSI | 0.813 | 0.306 | 0.329 | 0.746 | 0.458 | 0.93 | 0.808 | 0.413 |
| RAS | 0.492 | 0.449 | 0.424 | 0.528 | 0.572 | 0.311 | 0.733 |  |

Table 5. Surface under the cumulative ranking score of postoperative complications, range of motion, visual analogue scale scores, operative time, and total blood loss

|  | Postoperative complications | ROM | VAS scores | Operative time | Total blood loss |
| --- | --- | --- | --- | --- | --- |
| CON | 0.272 | 0.287 | 0.262 | 0.671 | 0.104 |
| CAS | 0.691 | 0.664 | 0.958 | 0.328 | 0.399 |
| PSI | 0.248 | 0.649 | 0.658 | 0.995 | 0.774 |
| RAS | 0.789 | 0.399 | 0.122 | 0.005 | 0.723 |

**Supplement 5:** **Forest plot of all outcomes**


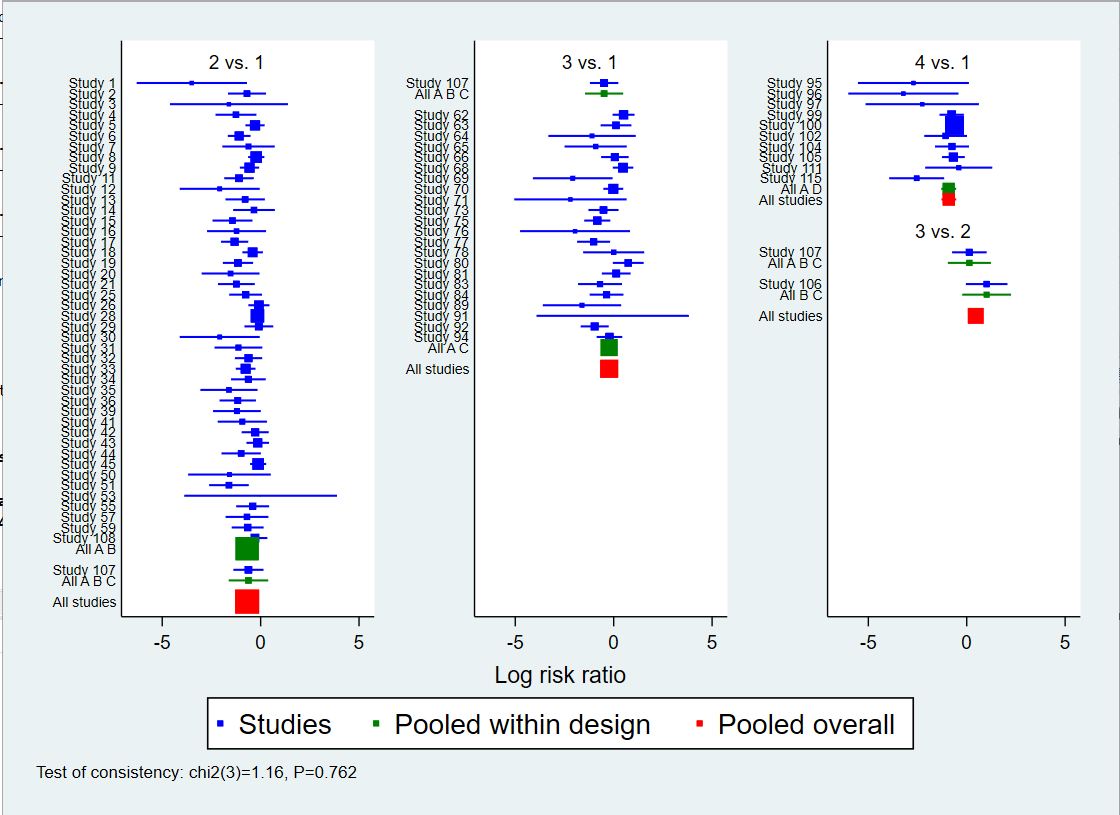


Fig. 5. Forest plot of mechanical axis outliers. 1 = conventional cutting instrument; 2 = computer-assisted navigation system; 3 = patient-specific instrument; 4 = robot-assisted system.


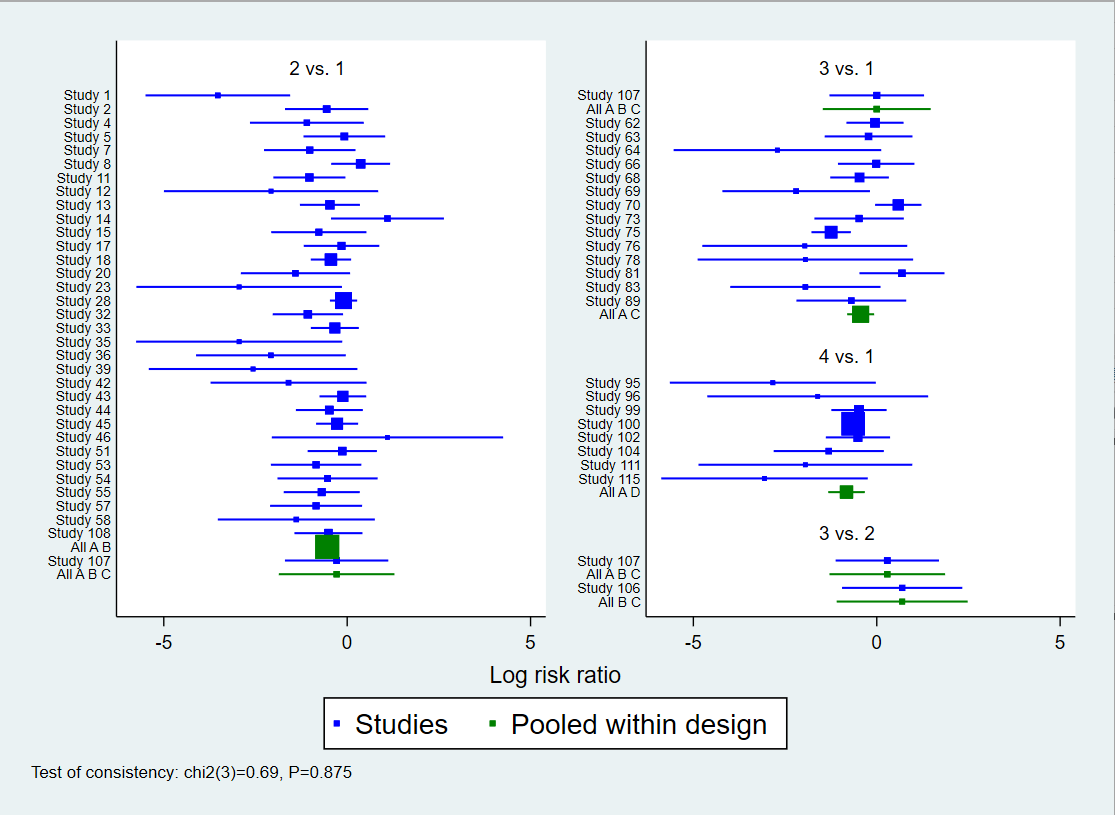


Fig. 6. Forest plot of coronal femoral component angle outliers. 1 = conventional cutting instrument; 2 = computer-assisted navigation system; 3 = patient-specific instrument; 4 = robot-assisted system.


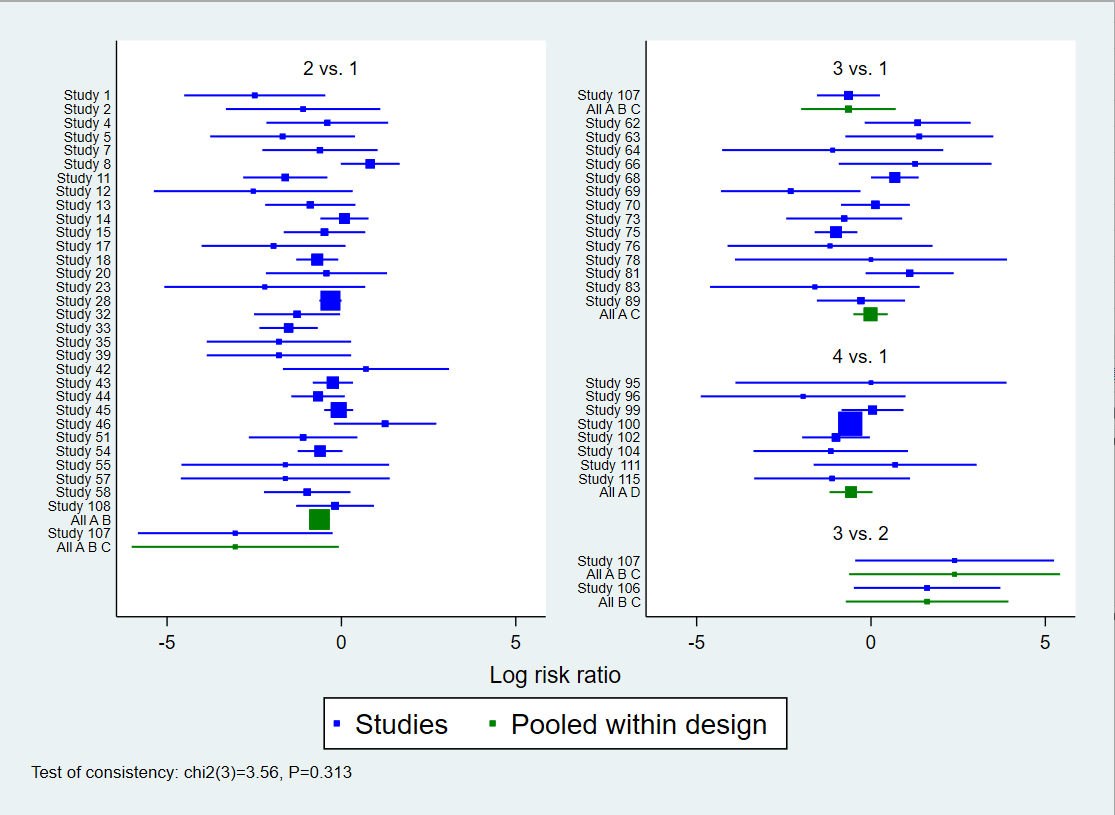


Fig. 7. Forest plot of coronal tibial component angle outliers. 1 = conventional cutting instrument; 2 = computer-assisted navigation system; 3 = patient-specific instrument; 4 = robot-assisted system.


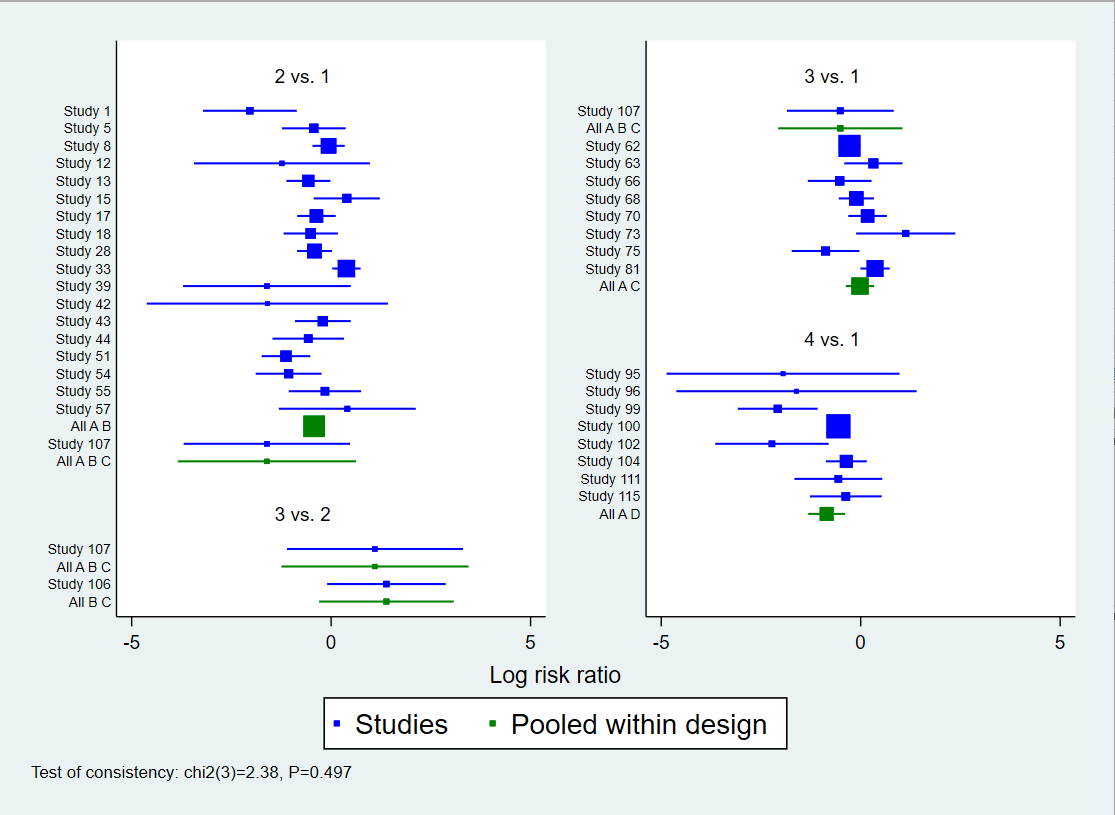


Fig. 8. Forest plot of sagittal femoral component angle outliers. 1 = conventional cutting instrument; 2 = computer-assisted navigation system; 3 = patient-specific instrument; 4 = robot-assisted system.


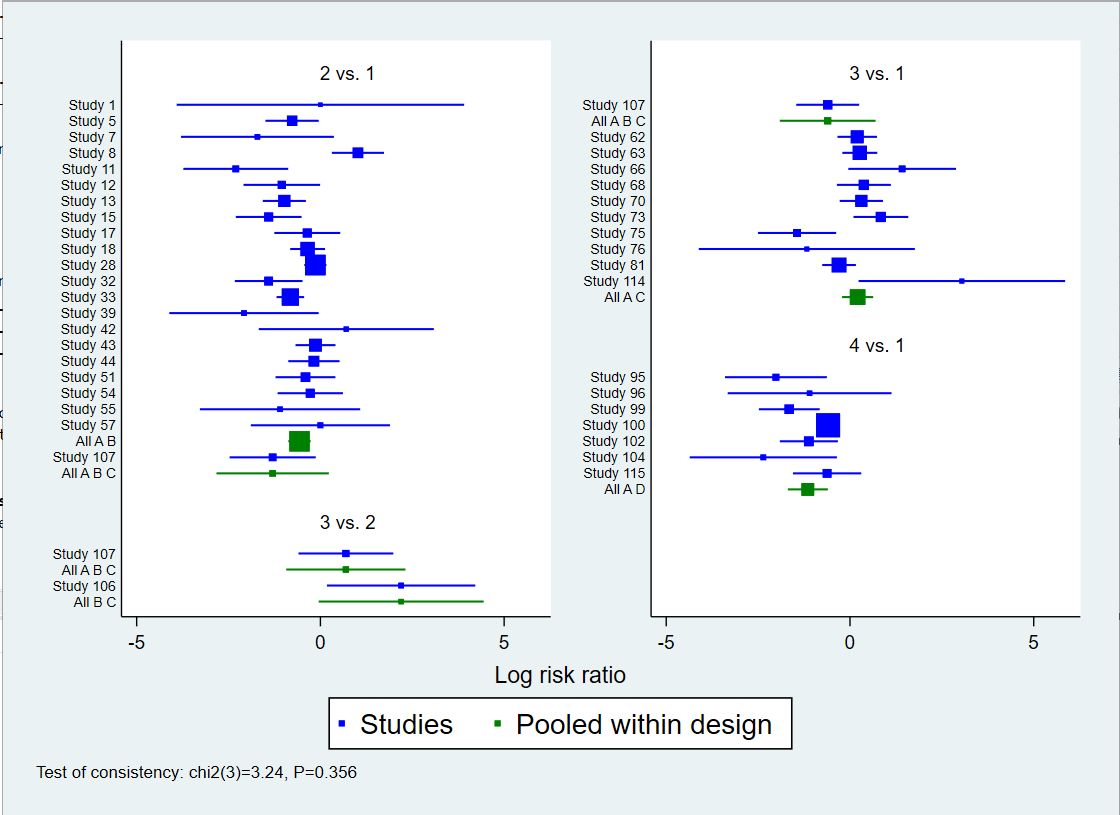


Fig. 9. Forest plot of sagittal tibial component angle outliers. 1 = conventional cutting instrument; 2 = computer-assisted navigation system; 3 = patient-specific instrument; 4 = robot-assisted system.


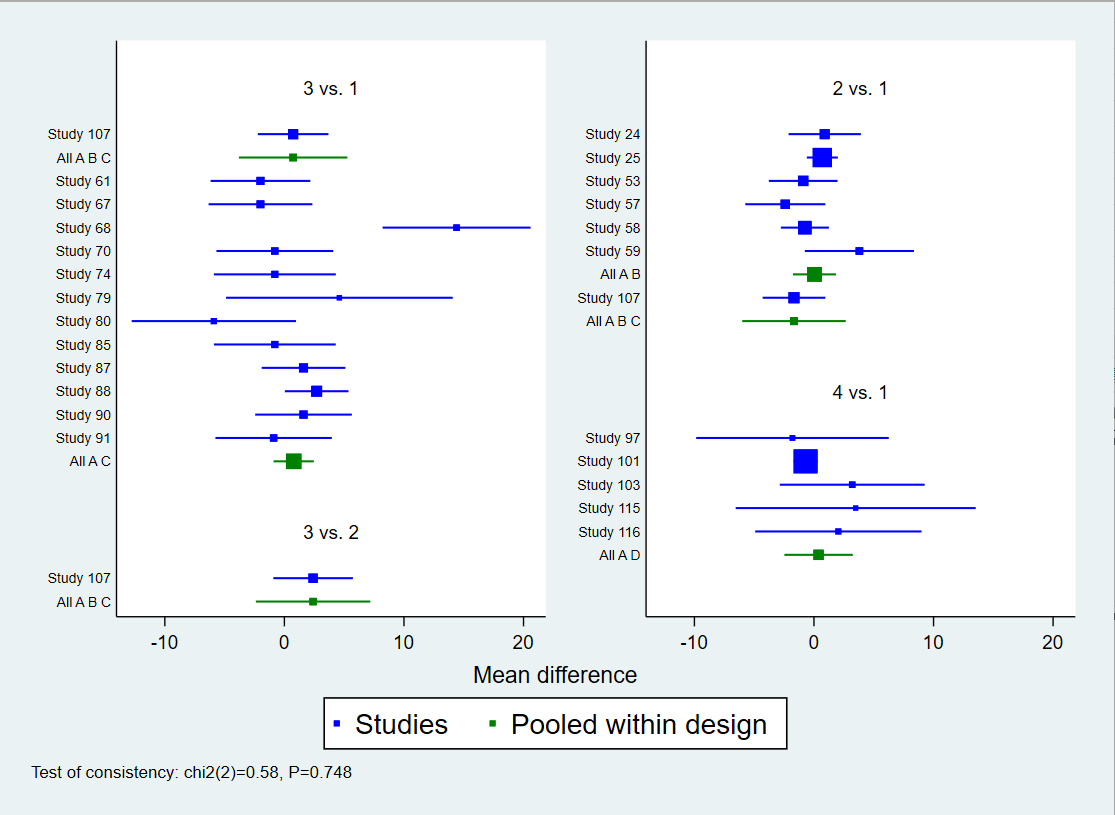


Fig. 10. Forest plot of short-term Knee Society Knee Scores. 1 = conventional cutting instrument; 2 = computer-assisted navigation system; 3 = patient-specific instrument; 4 = robot-assisted system.


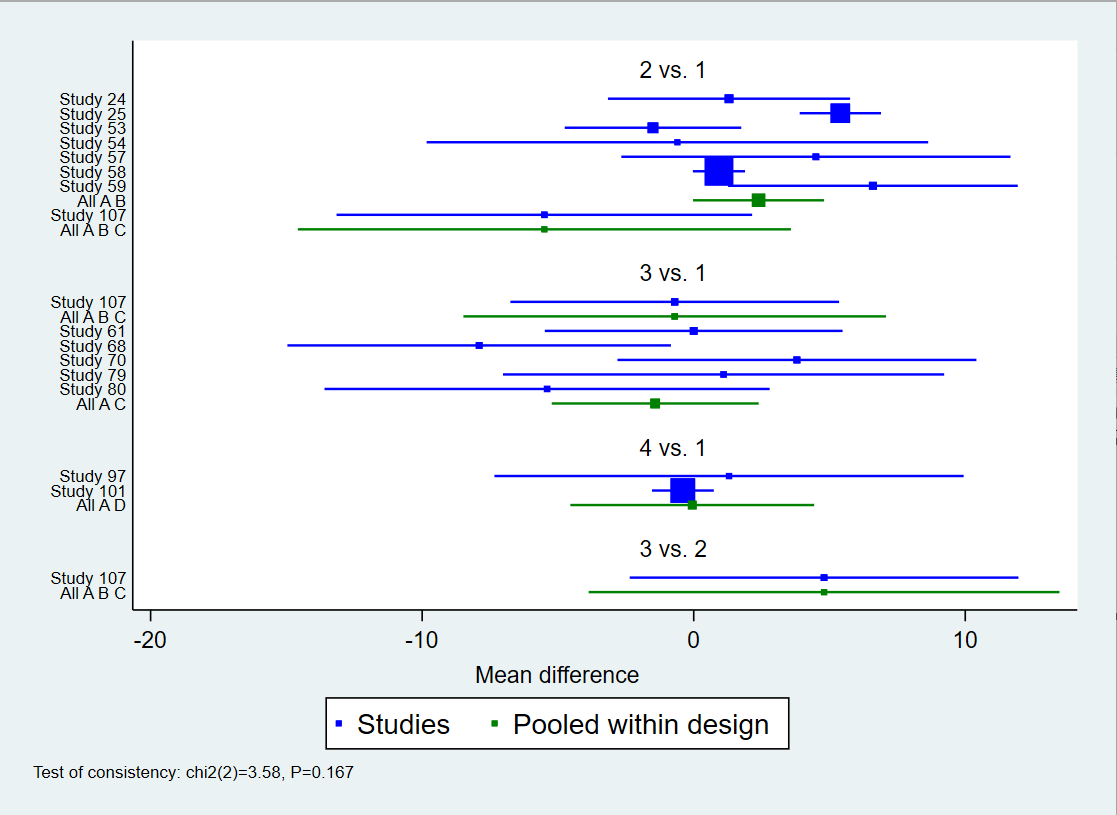


Fig. 11. Forest plot of short-term Knee Society Function Scores. 1 = conventional cutting instrument; 2 = computer-assisted navigation system; 3 = patient-specific instrument; 4 = robot-assisted system.


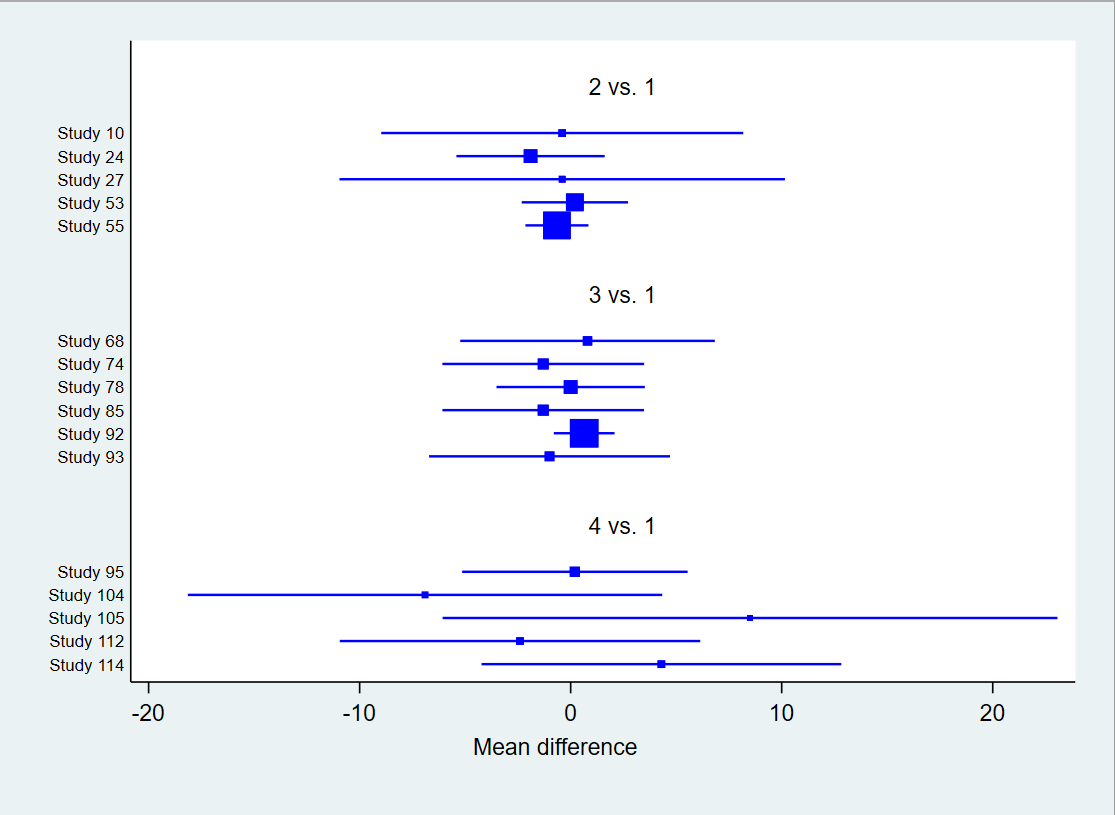


Fig. 12. Forest plot of short-term the Western Ontario and McMaster Universities scores. 1 = conventional cutting instrument; 2 = computer-assisted navigation system; 3 = patient-specific instrument; 4 = robot-assisted system.


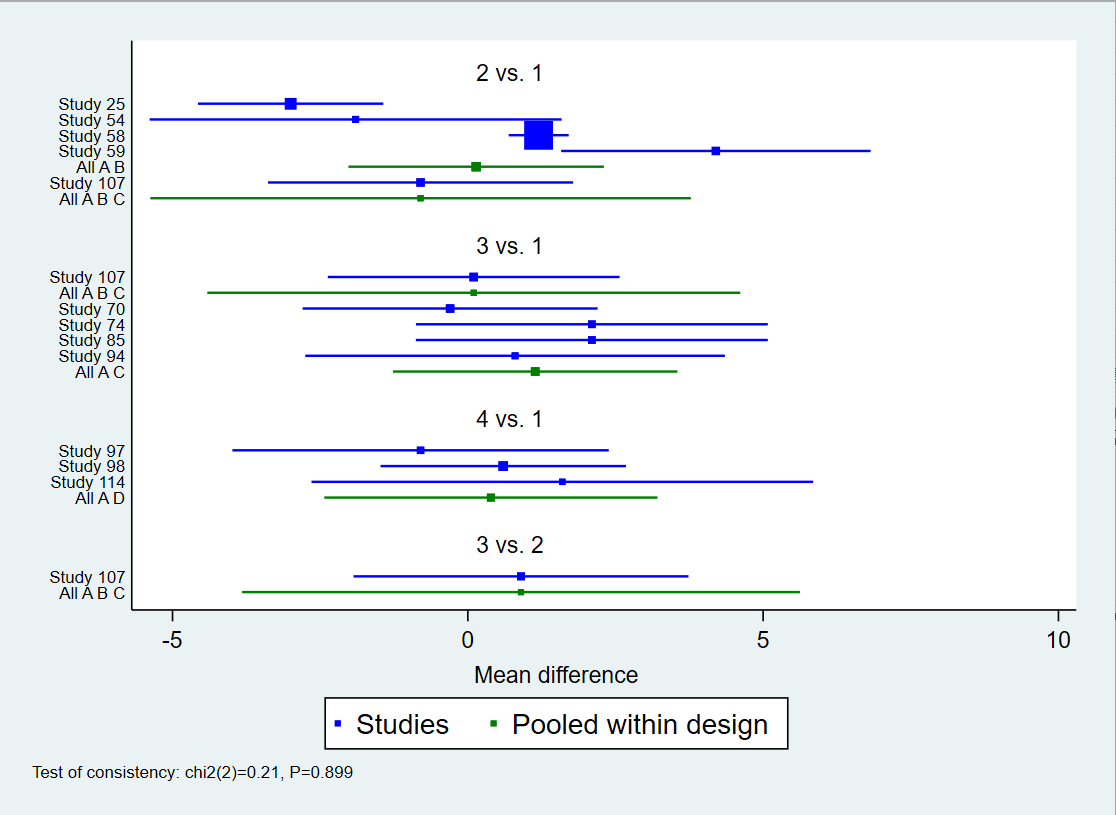


Fig. 13. Forest plot of short-term Oxford Knee Scores. 1 = conventional cutting instrument; 2 = computer-assisted navigation system; 3 = patient-specific instrument; 4 = robot-assisted system.


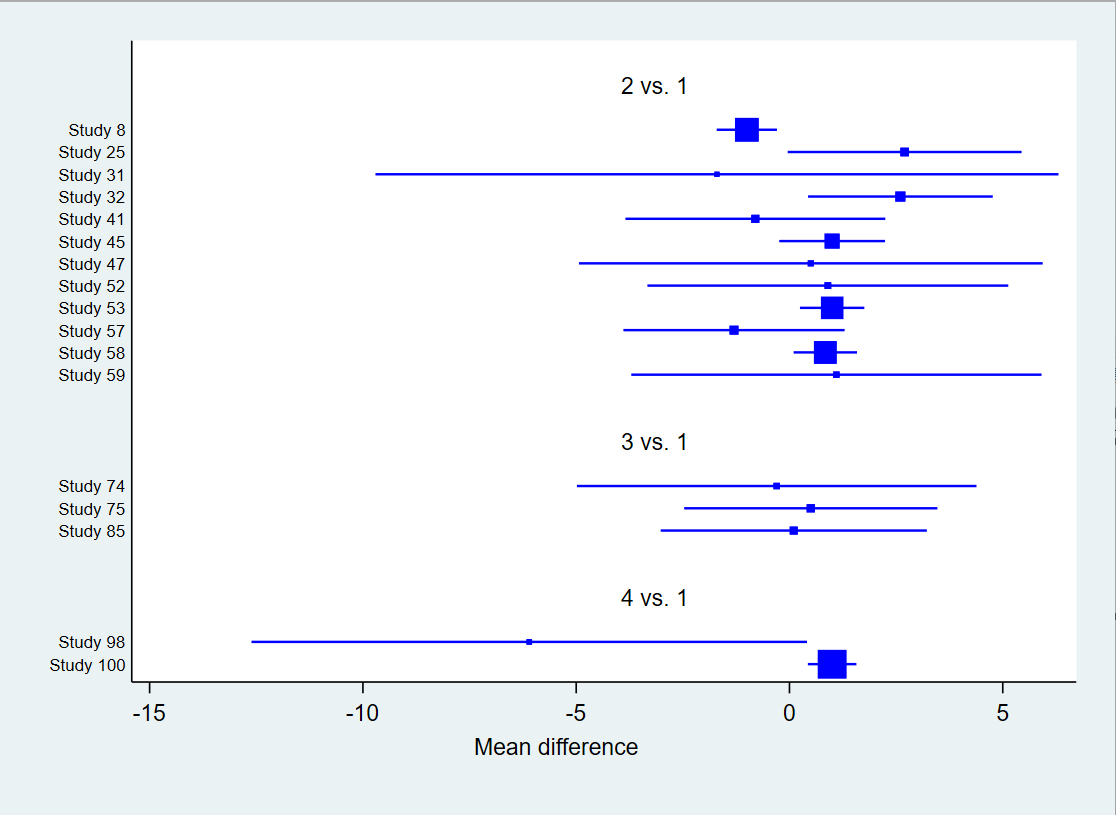


Fig. 14. Forest plot of medium-and-long-term Knee Society Knee Scores. 1 = conventional cutting instrument; 2 = computer-assisted navigation system; 3 = patient-specific instrument; 4 = robot-assisted system.


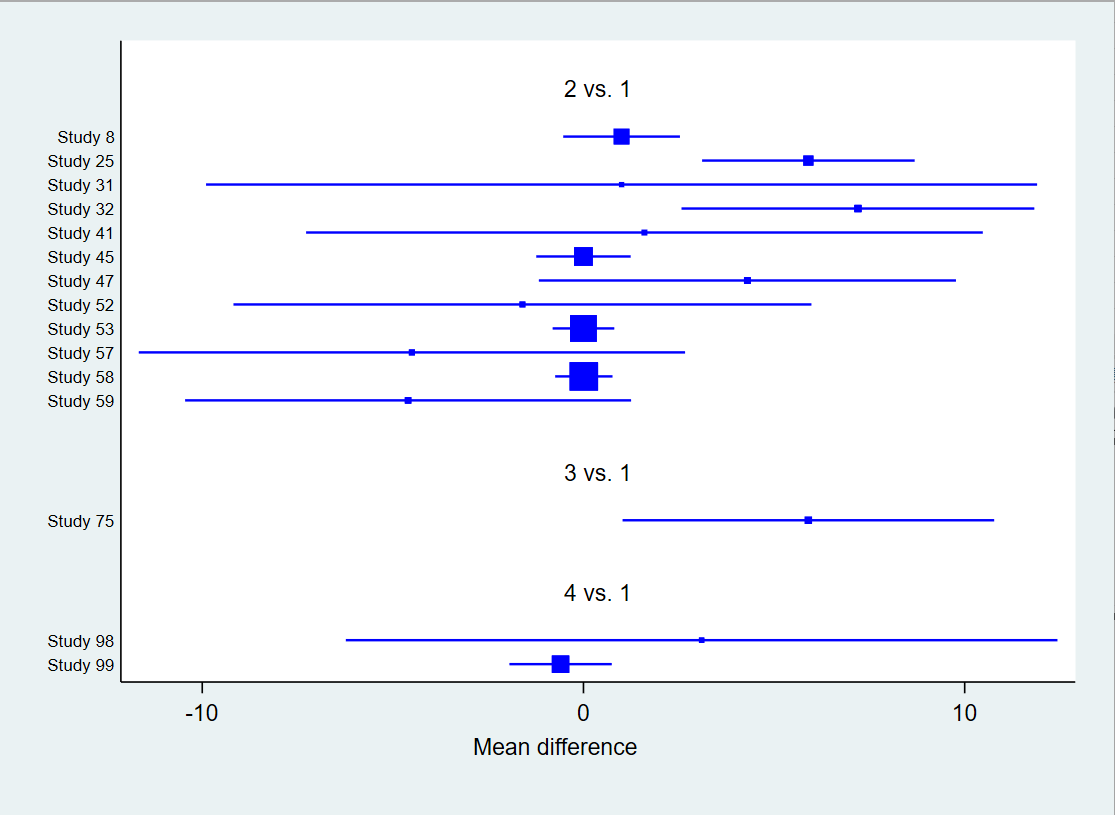


Fig. 15. Forest plot of medium-and-long-term Knee Society Function Scores. 1 = conventional cutting instrument; 2 = computer-assisted navigation system; 3 = patient-specific instrument; 4 = robot-assisted system.


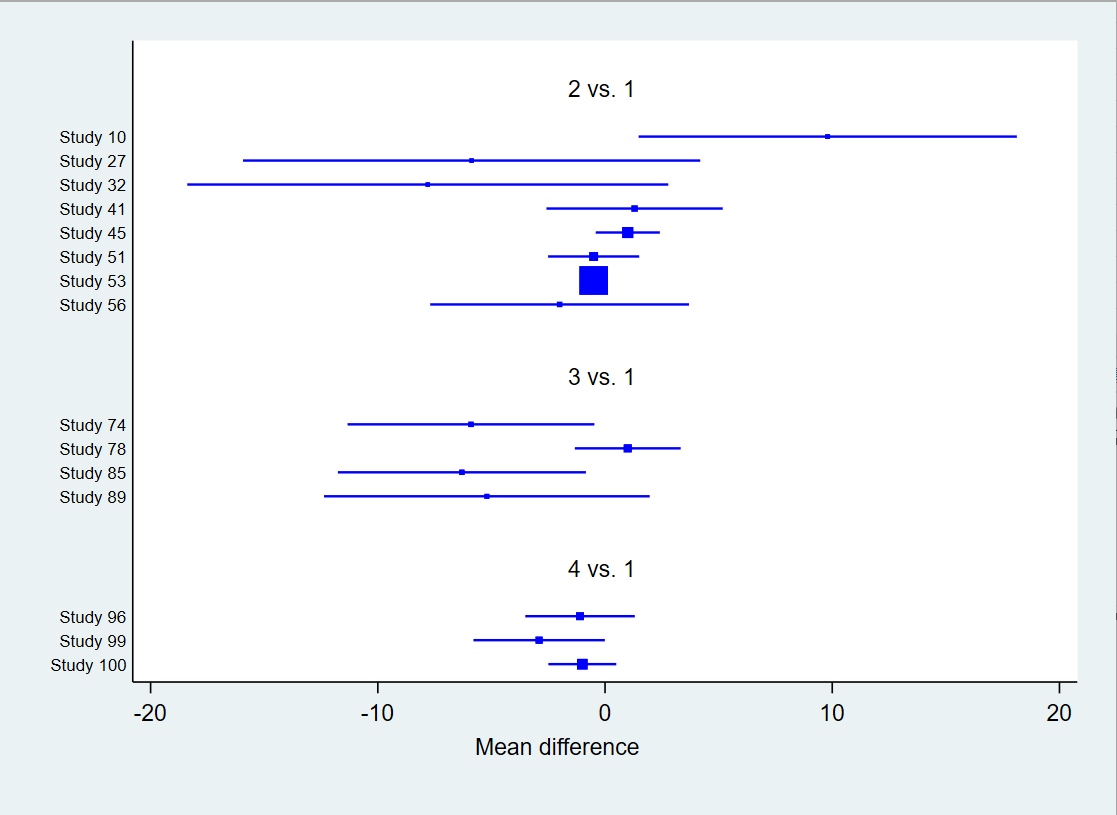


Fig. 16. Forest plot of medium-and-long -term the Western Ontario and McMaster Universities scores. 1 = conventional cutting instrument; 2 = computer-assisted navigation system; 3 = patient-specific instrument; 4 = robot-assisted system.


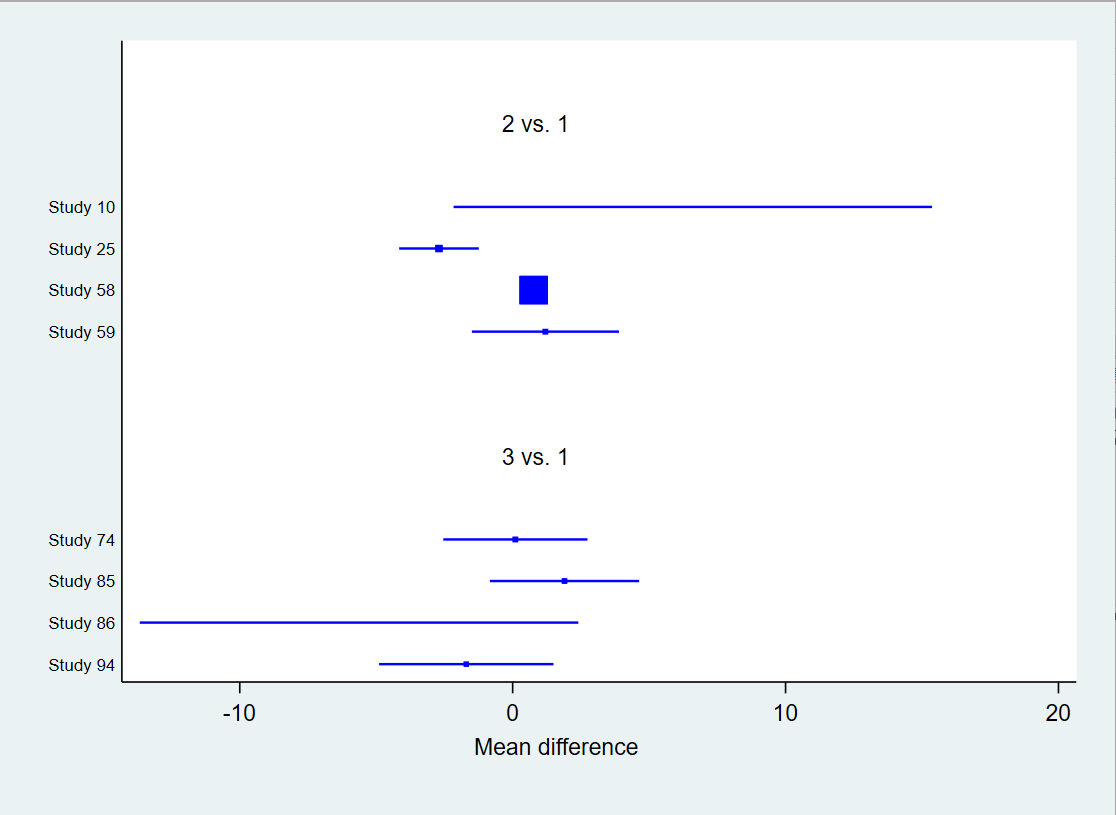


Fig. 17. Forest plot of medium-and-long-term Oxford Knee Scores. 1 = conventional cutting instrument; 2 = computer-assisted navigation system; 3 = patient-specific instrument.


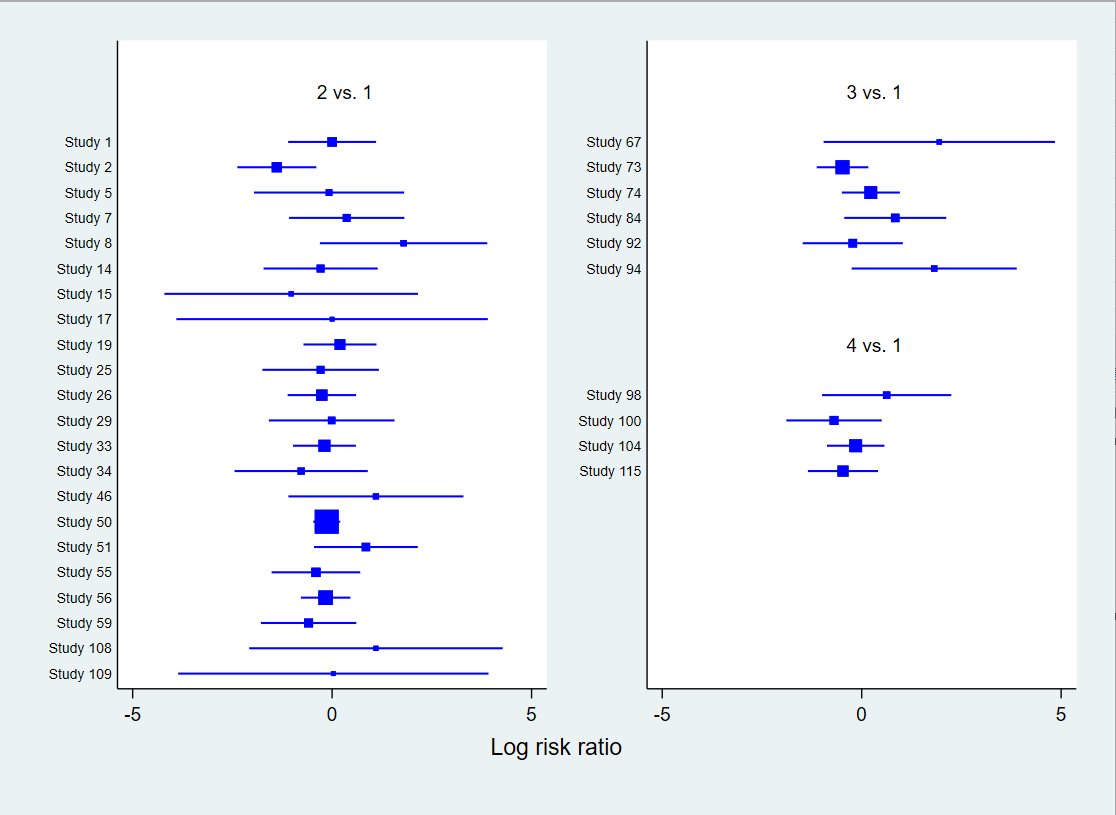


Fig. 18. Forest plot of postoperative complications. 1 = conventional cutting instrument; 2 = computer-assisted navigation system; 3 = patient-specific instrument; 4 = robot-assisted system.


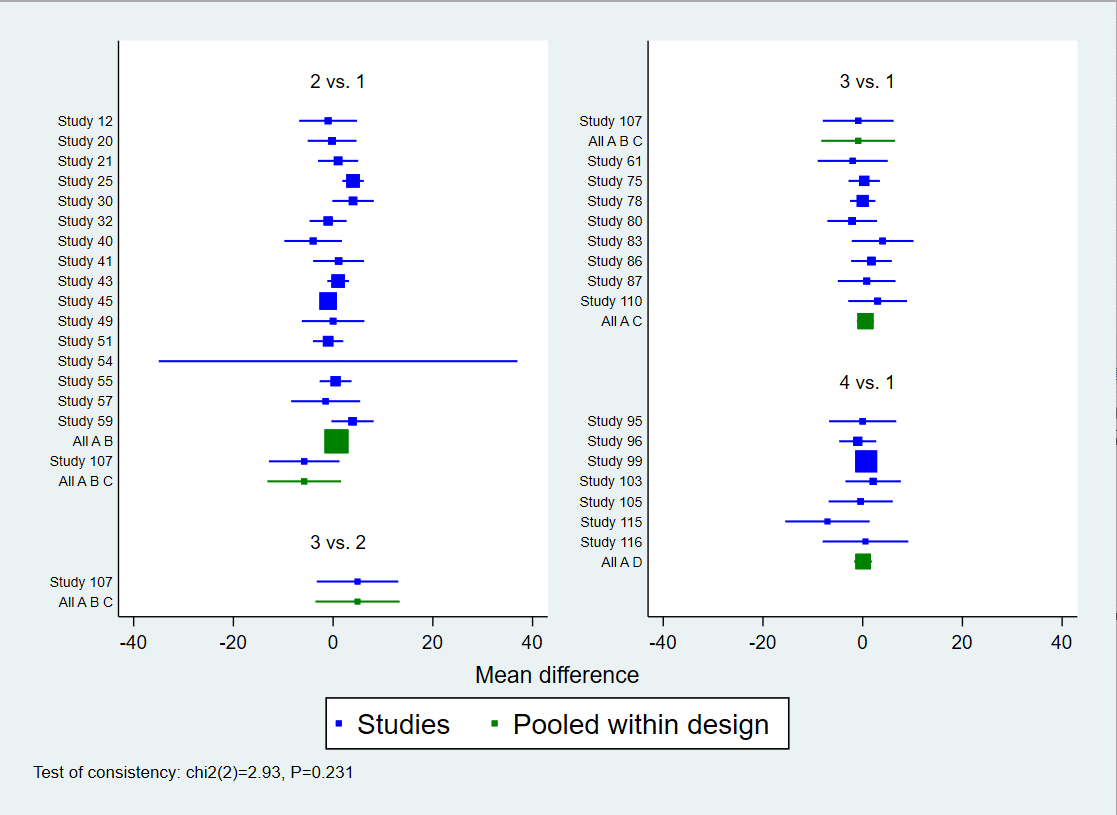


Fig. 19. Forest plot of range of motion. 1 = conventional cutting instrument; 2 = computer-assisted navigation system; 3 = patient-specific instrument; 4 = robot-assisted system.


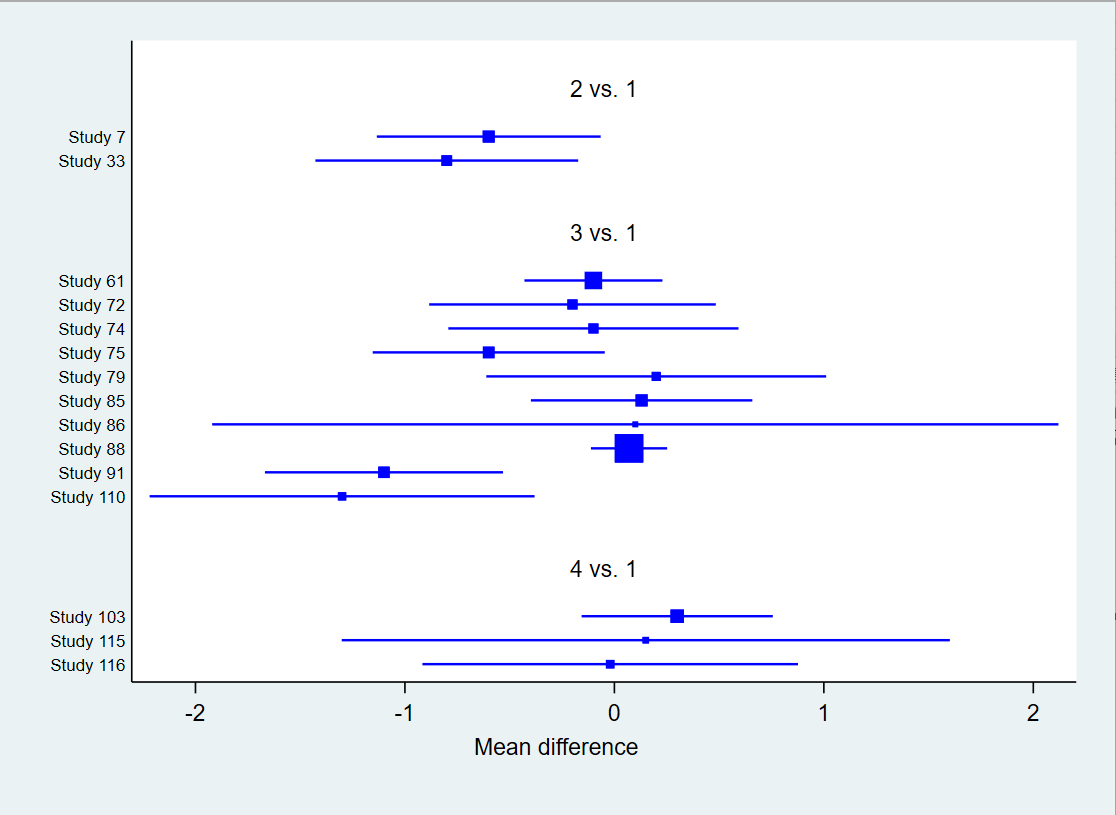


Fig. 20. Forest plot of visual analogue scale scores. 1 = conventional cutting instrument; 2 = computer-assisted navigation system; 3 = patient-specific instrument; 4 = robot-assisted system.


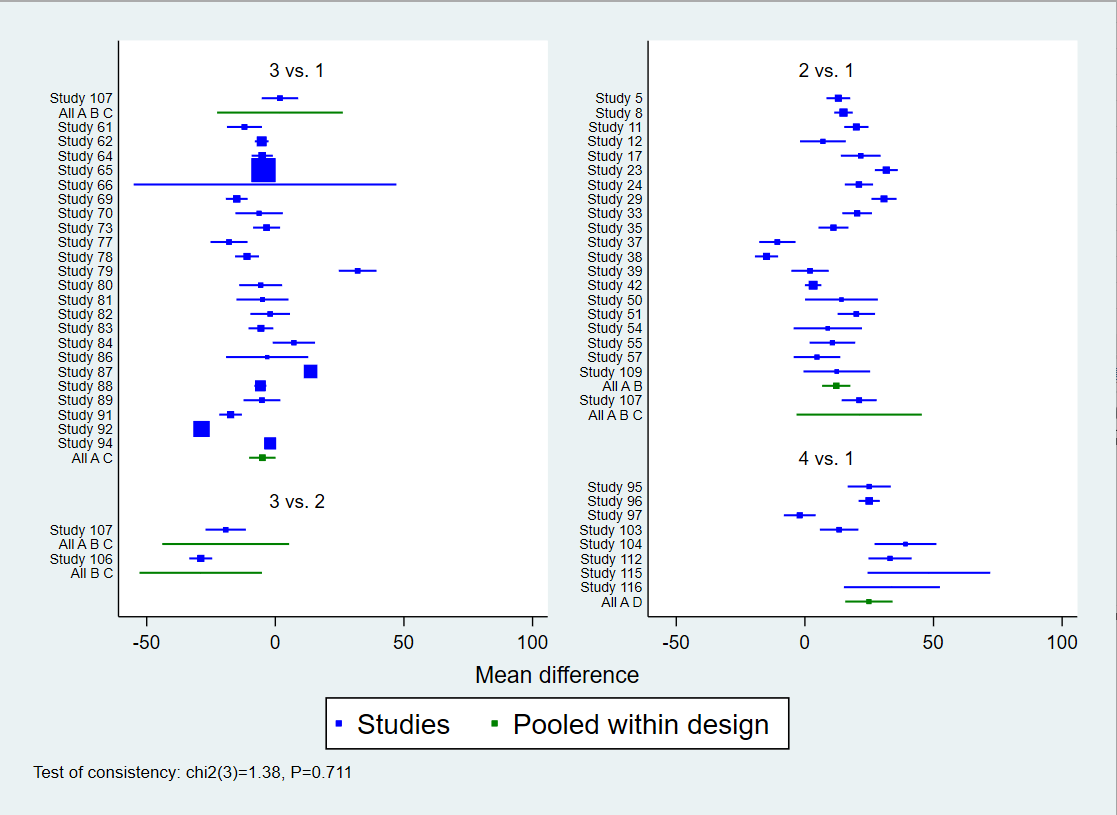


Fig. 21. Forest plot of operative time. 1 = conventional cutting instrument; 2 = computer-assisted navigation system; 3 = patient-specific instrument; 4 = robot-assisted system.


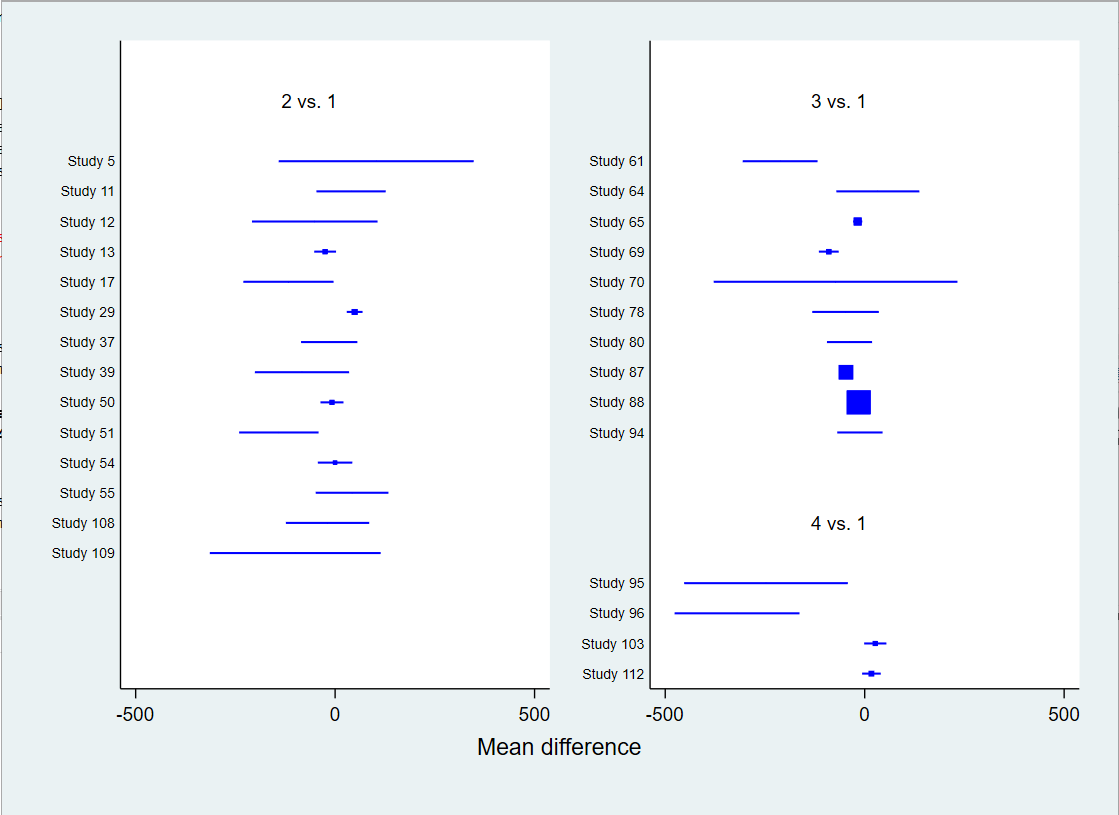


Fig. 22. Forest plot of total blood loss. 1 = conventional cutting instrument; 2 = computer-assisted navigation system; 3 = patient-specific instrument; 4 = robot-assisted system.

**Supplement 6:** **Funnel plot of all outcomes**


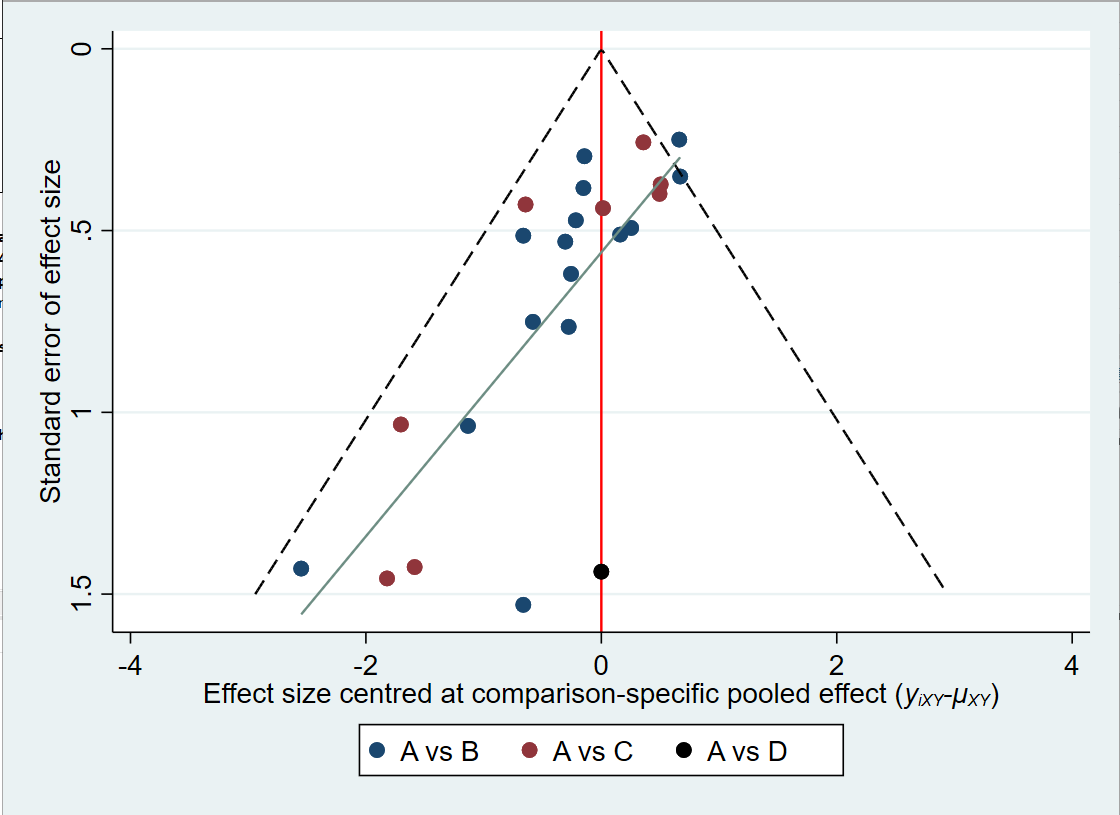


Fig. 23. Funnel plot of mechanical axis outliers. A = conventional cutting instrument; B = computer-assisted navigation system; C = patient-specific instrument; D = robot-assisted system.


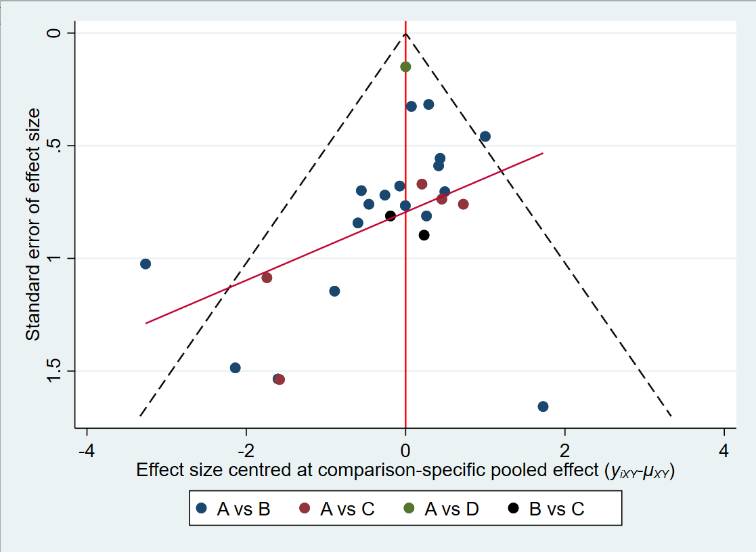


Fig. 24. Funnel plot of coronal femoral component angle outliers. A = conventional cutting instrument; B = computer-assisted navigation system; C = patient-specific instrument; D = robot-assisted system.


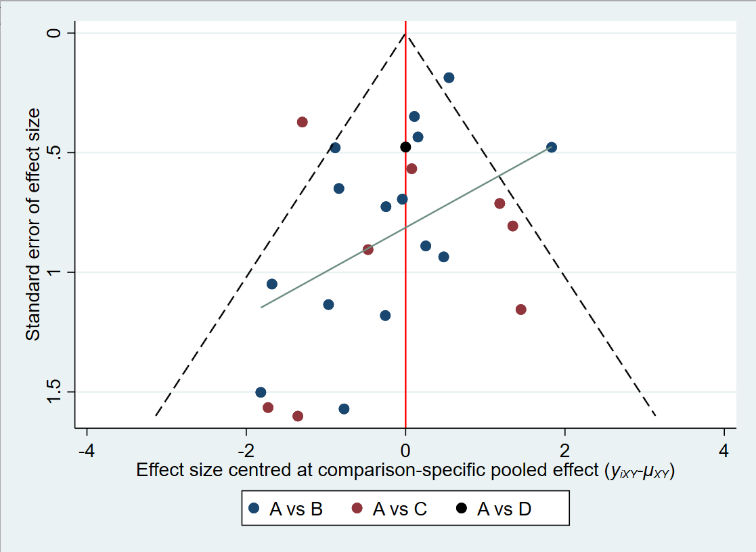


Fig. 25. Funnel plot of coronal tibial component angle outliers. A = conventional cutting instrument; B = computer-assisted navigation system; C = patient-specific instrument; D = robot-assisted system.


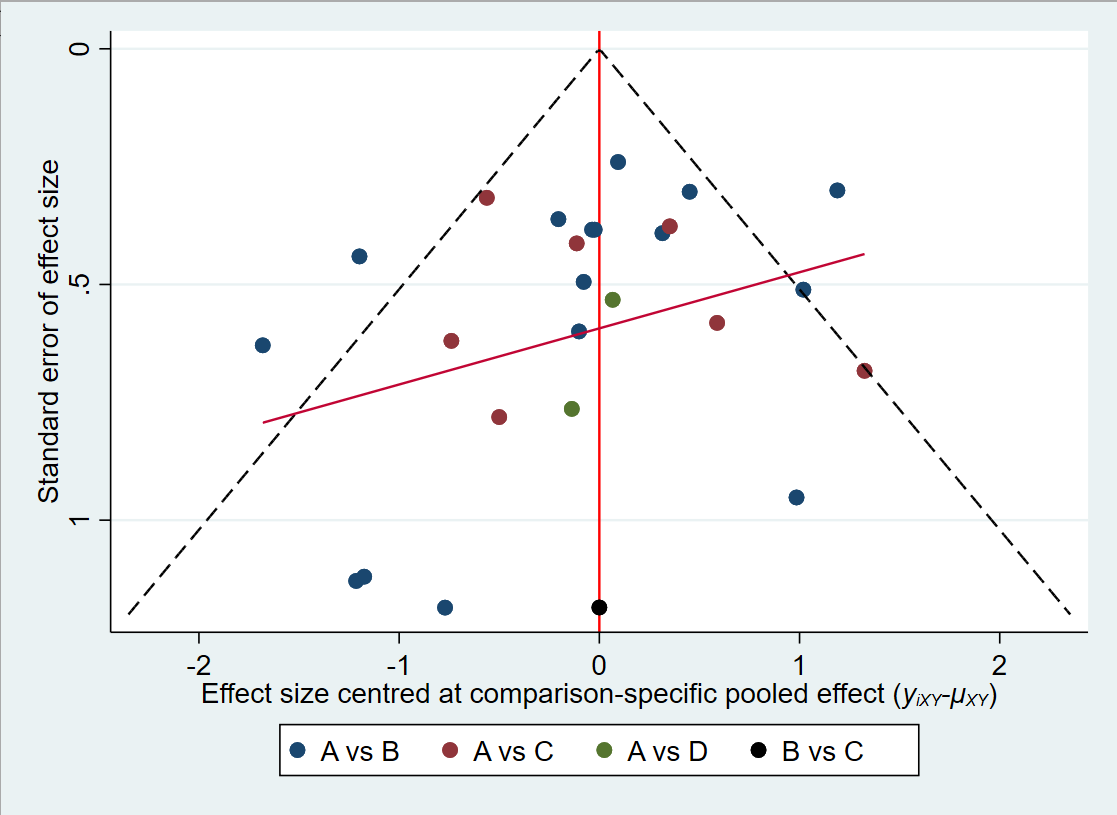


Fig. 26. Funnel plot of sagittal femoral component angle outliers. A = conventional cutting instrument; B = computer-assisted navigation system; C = patient-specific instrument; D = robot-assisted system.


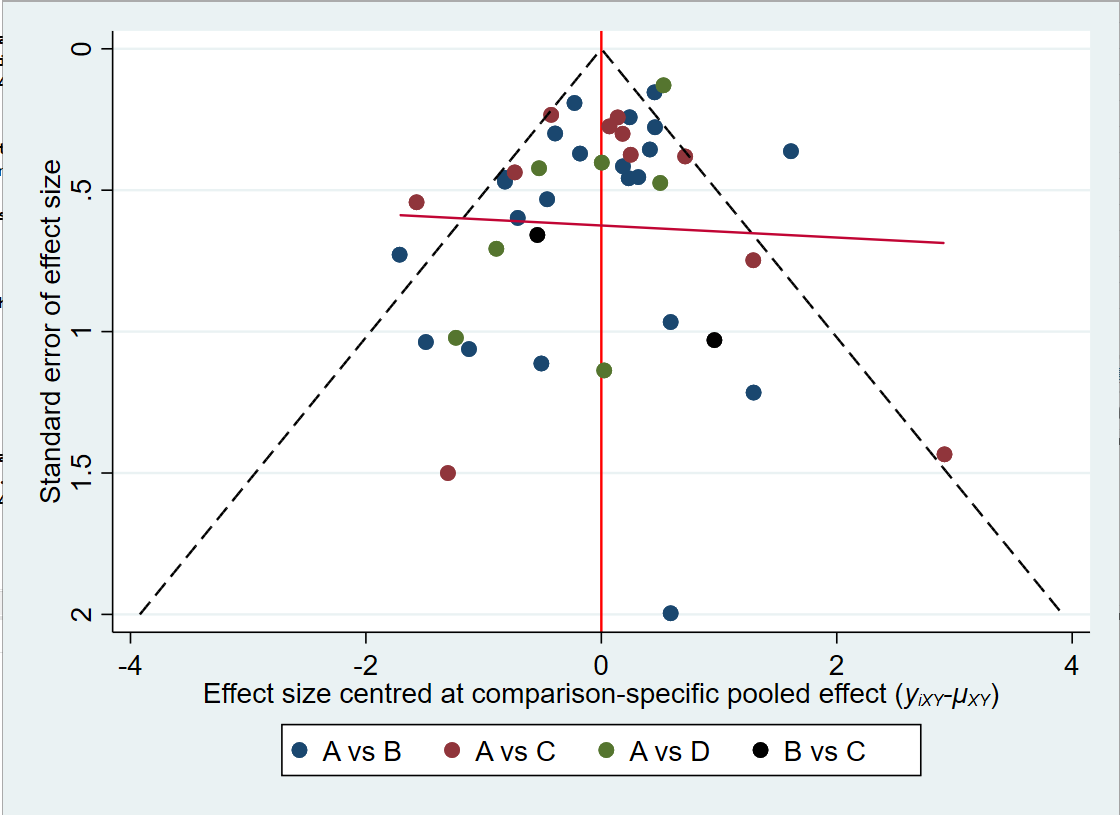


Fig. 27. Funnel plot of sagittal tibial component angle outliers. A = conventional cutting instrument; B = computer-assisted navigation system; C = patient-specific instrument; D = robot-assisted system.


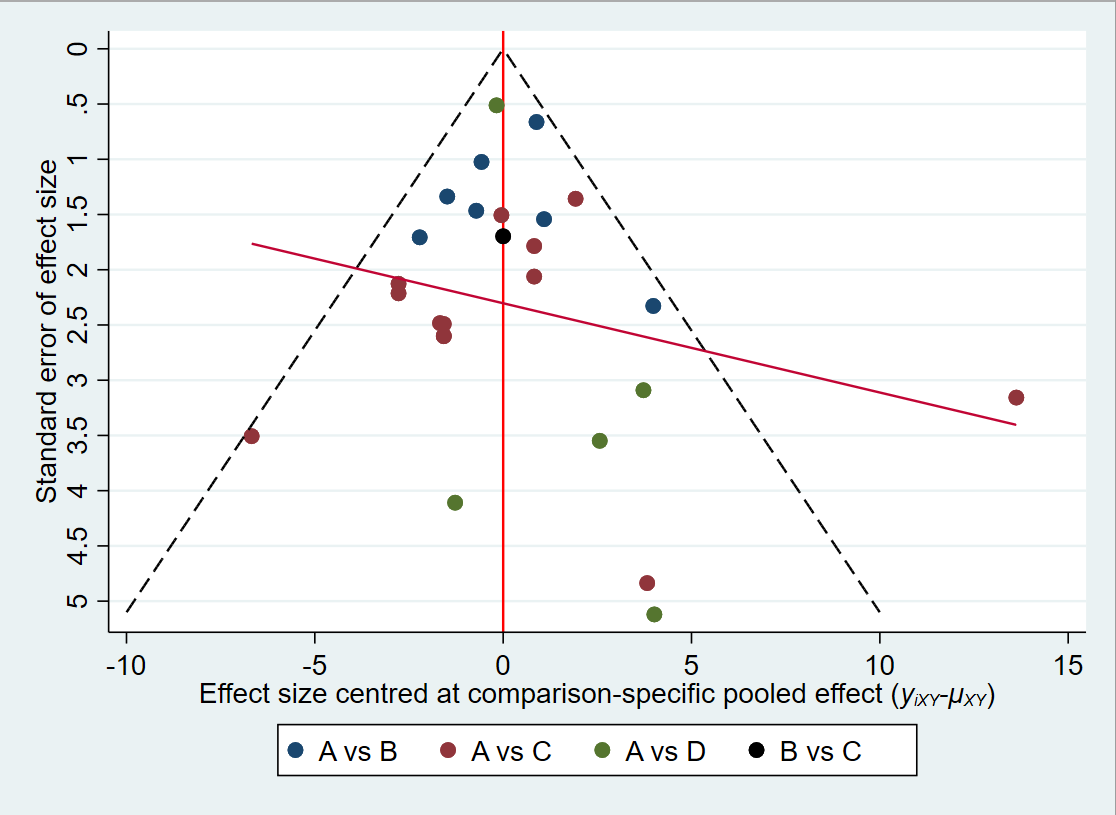


Fig. 28. Funnel plot of short-term Knee Society Knee Scores. A = conventional cutting instrument; B = computer-assisted navigation system; C = patient-specific instrument; D = robot-assisted system.


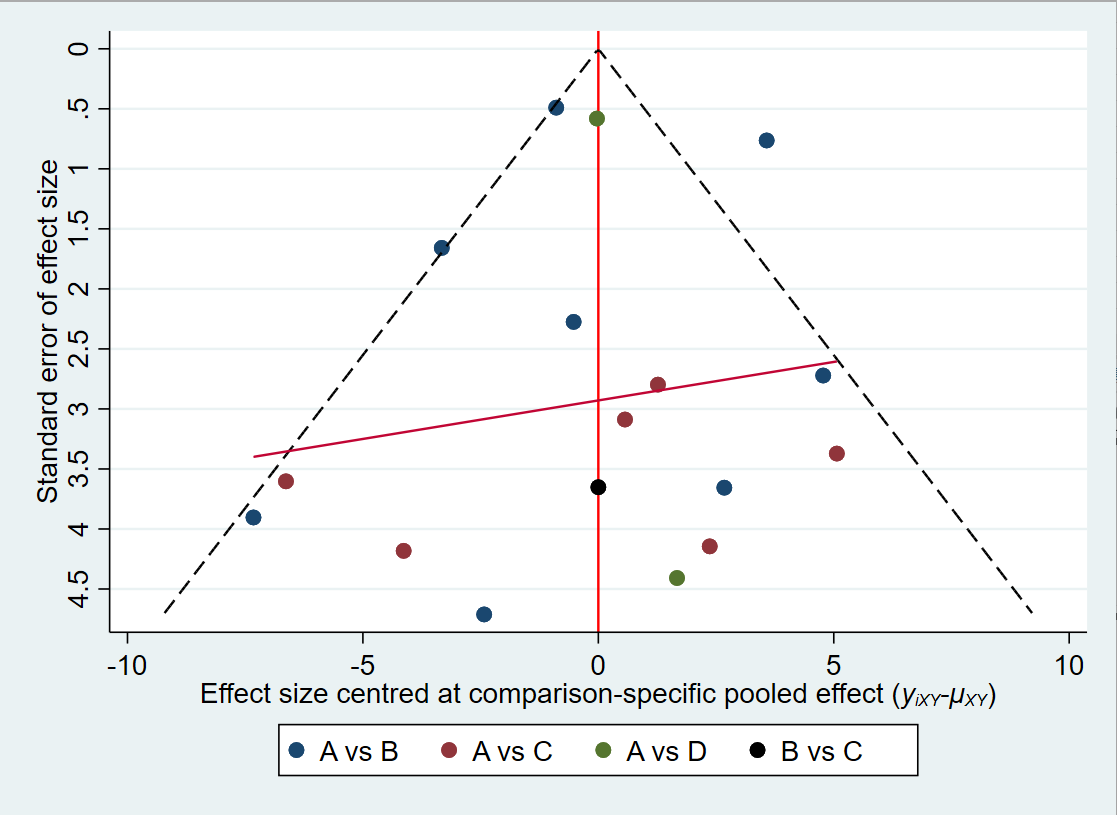


Fig. 29. Funnel plot of short-term Knee Society Function Scores. A = conventional cutting instrument; B = computer-assisted navigation system; C = patient-specific instrument; D = robot-assisted system.


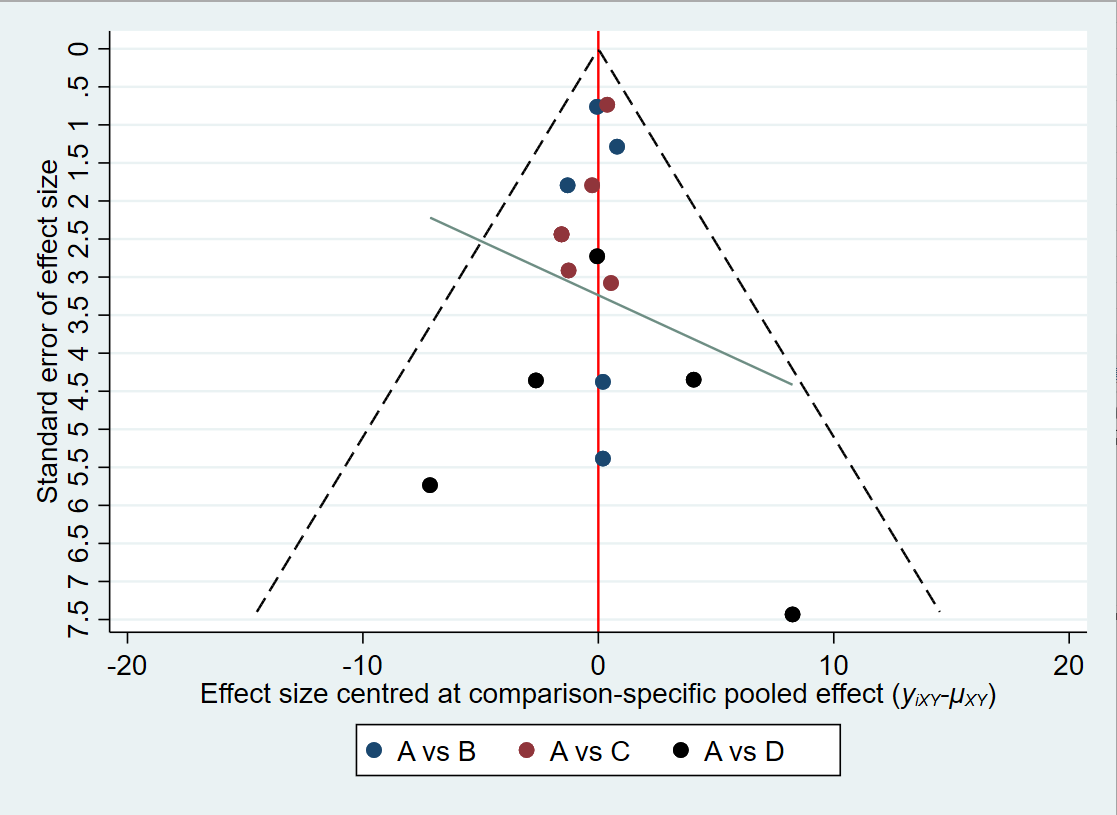


Fig. 30. Funnel plot of short-term the Western Ontario and McMaster Universities scores. A = conventional cutting instrument; B = computer-assisted navigation system; C = patient-specific instrument; D = robot-assisted system.


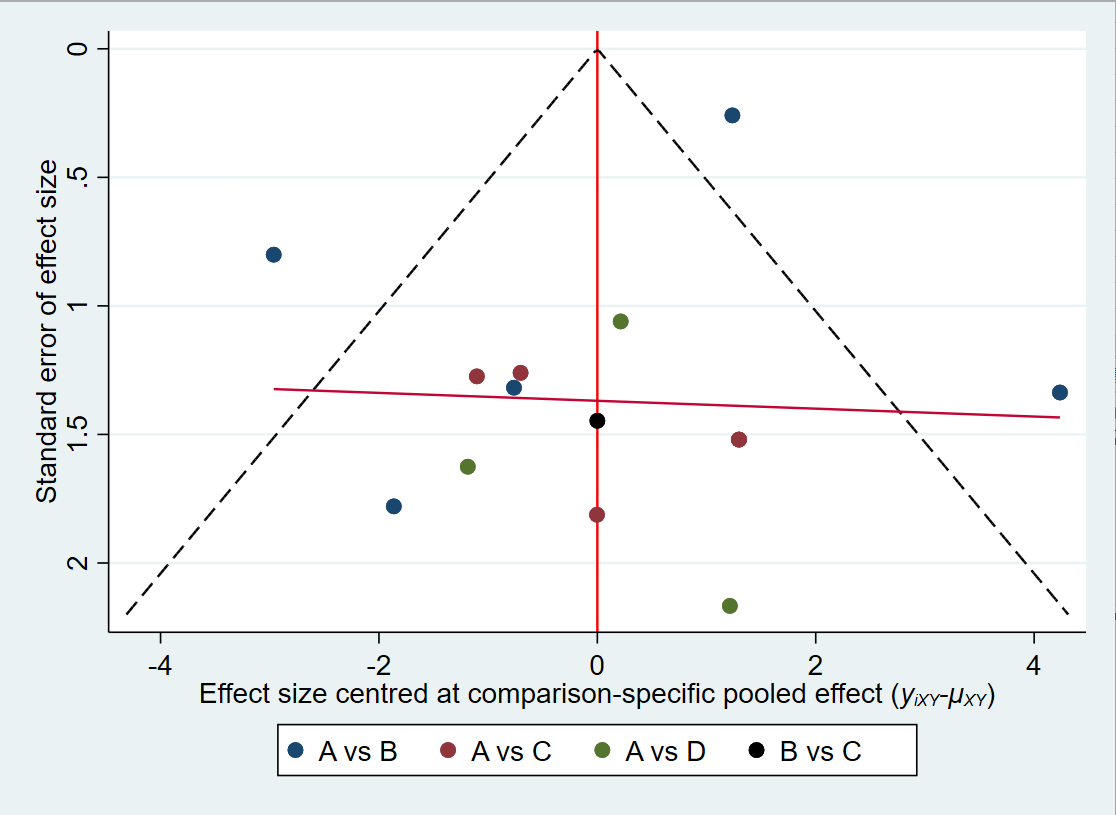


Fig. 31. Funnel plot of short-term Oxford Knee Scores. A = conventional cutting instrument; B = computer-assisted navigation system; C = patient-specific instrument; D = robot-assisted system.


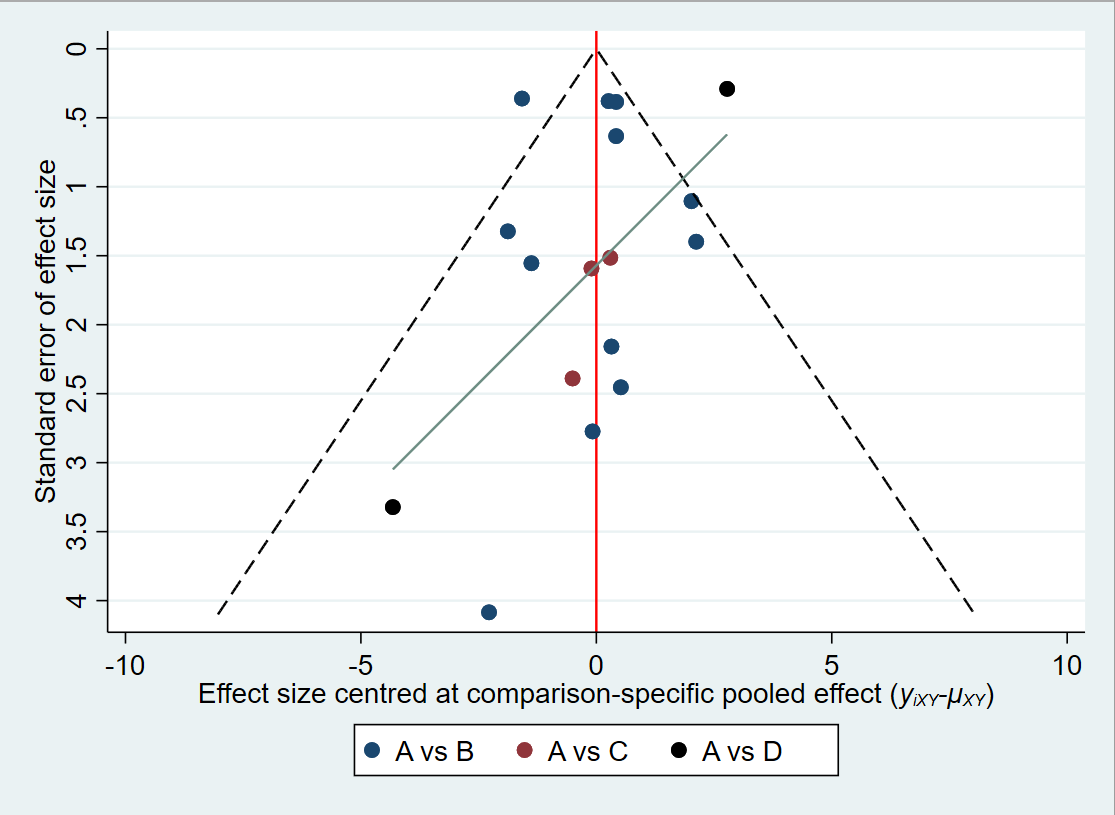


Fig. 32. Funnel plot of medium-and-long-term Knee Society Knee Scores. A = conventional cutting instrument; B = computer-assisted navigation system; C = patient-specific instrument; D = robot-assisted system.


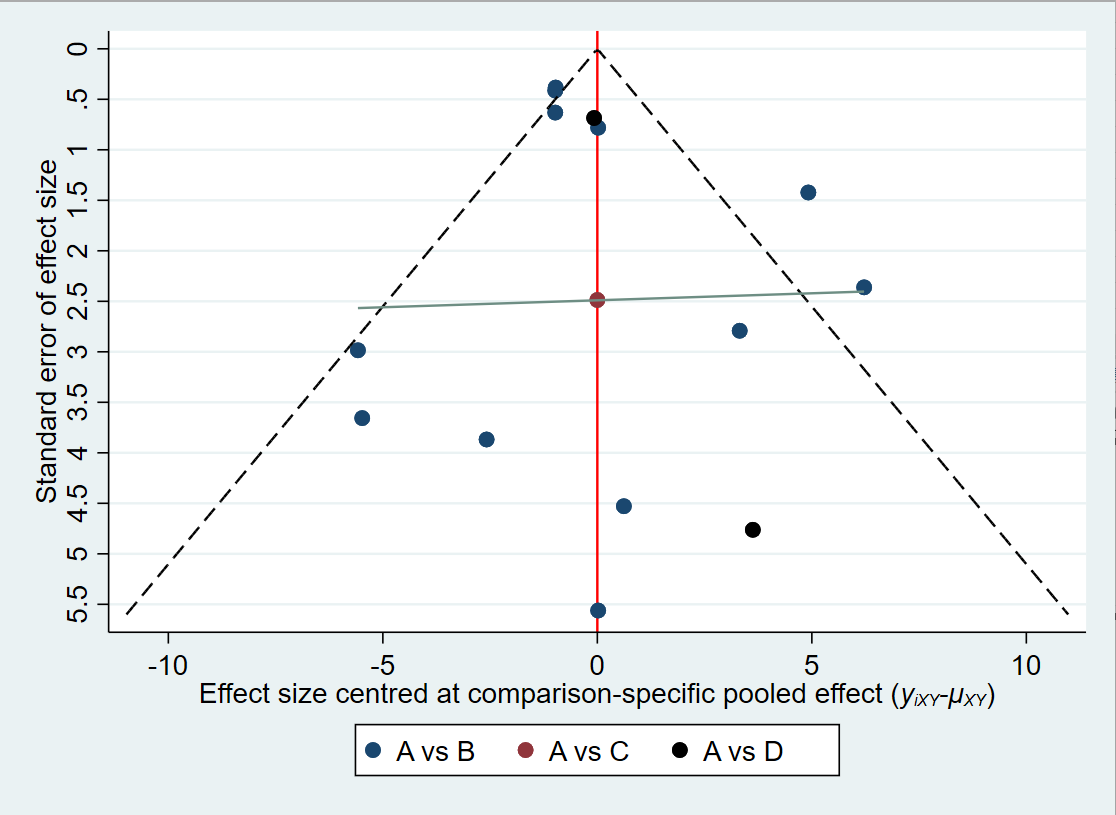


Fig. 33. Funnel plot of medium-and-long-term Knee Society Function Scores. A = conventional cutting instrument; B = computer-assisted navigation system; C = patient-specific instrument; D = robot-assisted system.


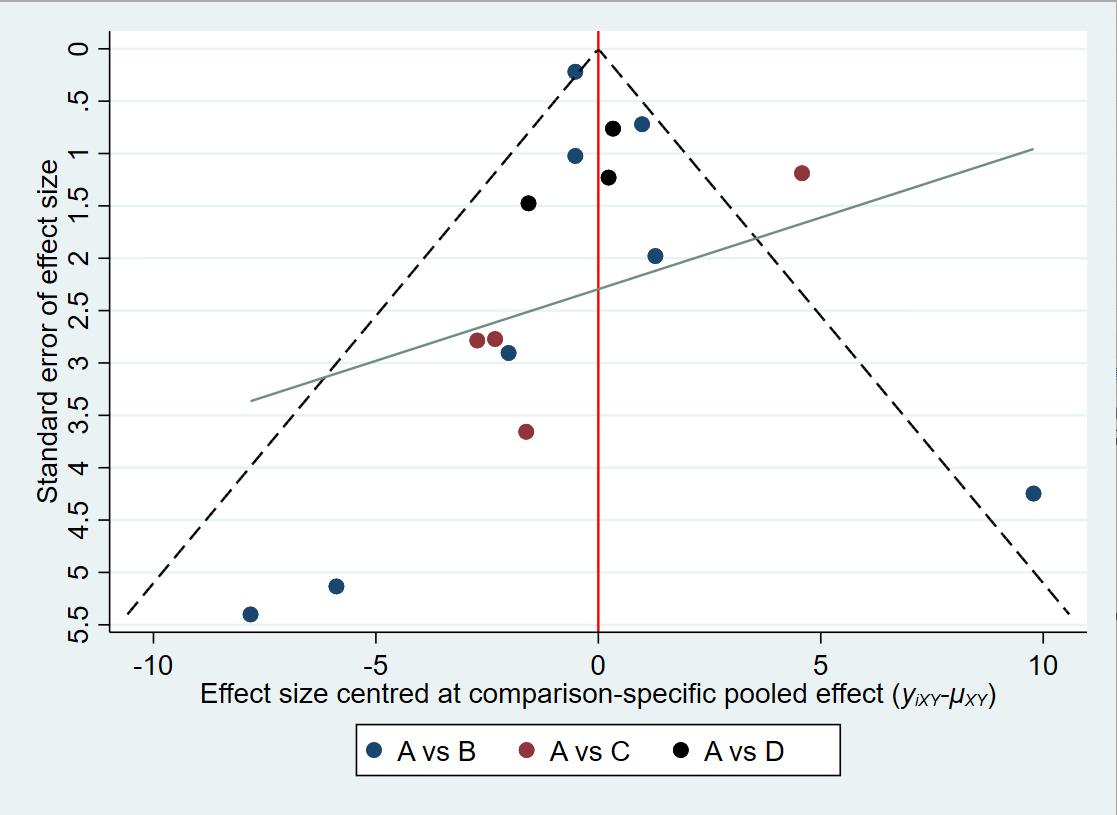


Fig. 34. Funnel plot of medium-and-long-term the Western Ontario and McMaster Universities scores. A = conventional cutting instrument; B = computer-assisted navigation system; C = patient-specific instrument; D = robot-assisted system.


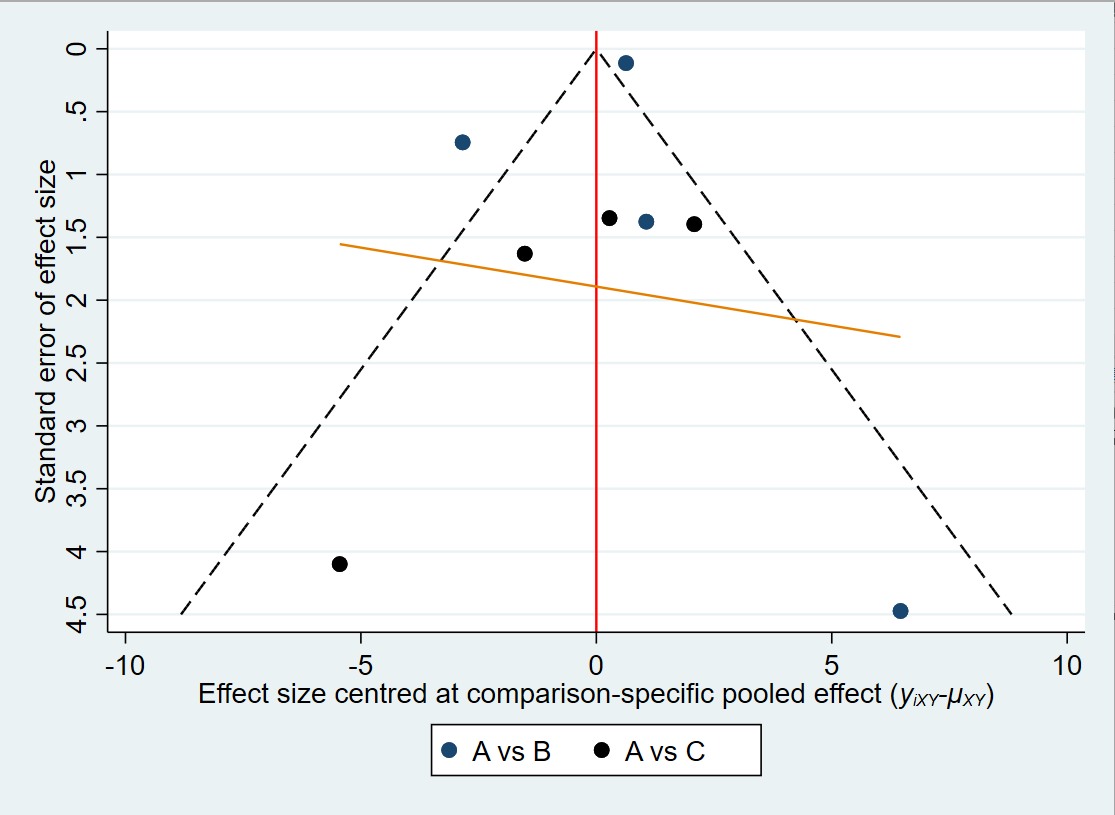


Fig. 35. Funnel plot of medium-and-long-term Oxford Knee Scores. A = conventional cutting instrument; B = computer-assisted navigation system; C = patient-specific instrument; D = robot-assisted system.


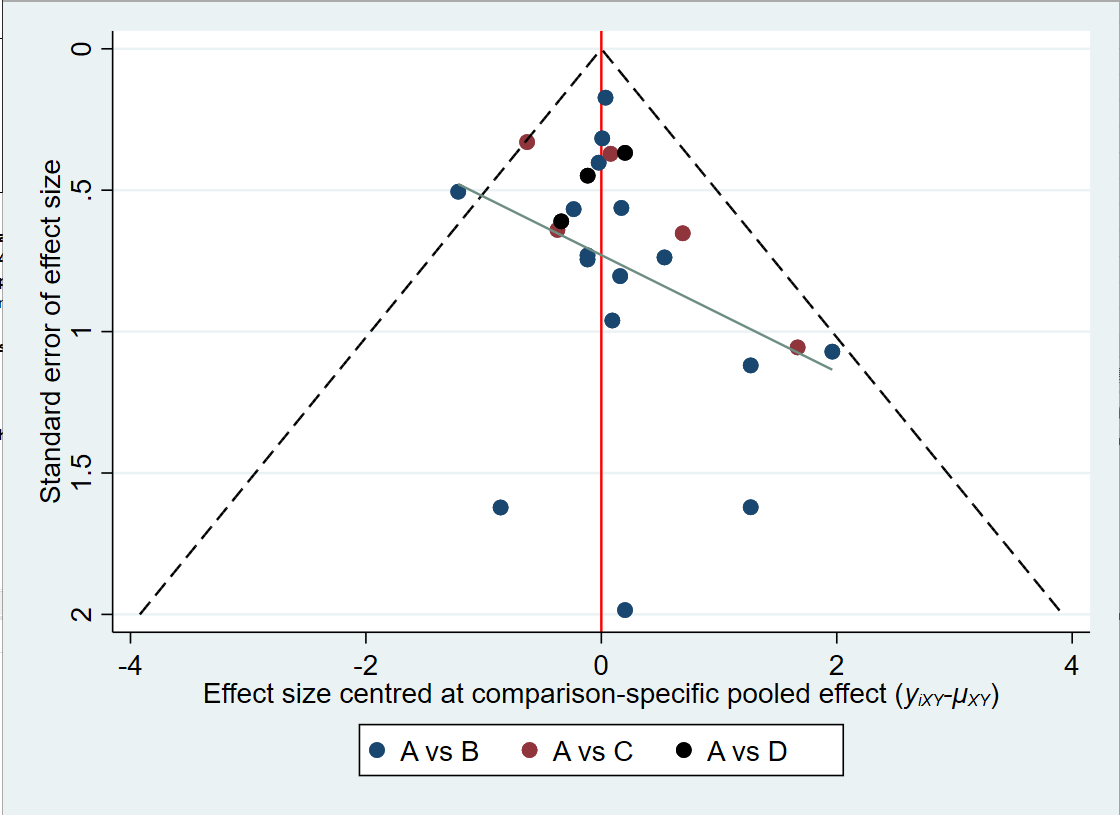


Fig. 36. Funnel plot of postoperative complications. A = conventional cutting instrument; B = computer-assisted navigation system; C = patient-specific instrument; D = robot-assisted system.


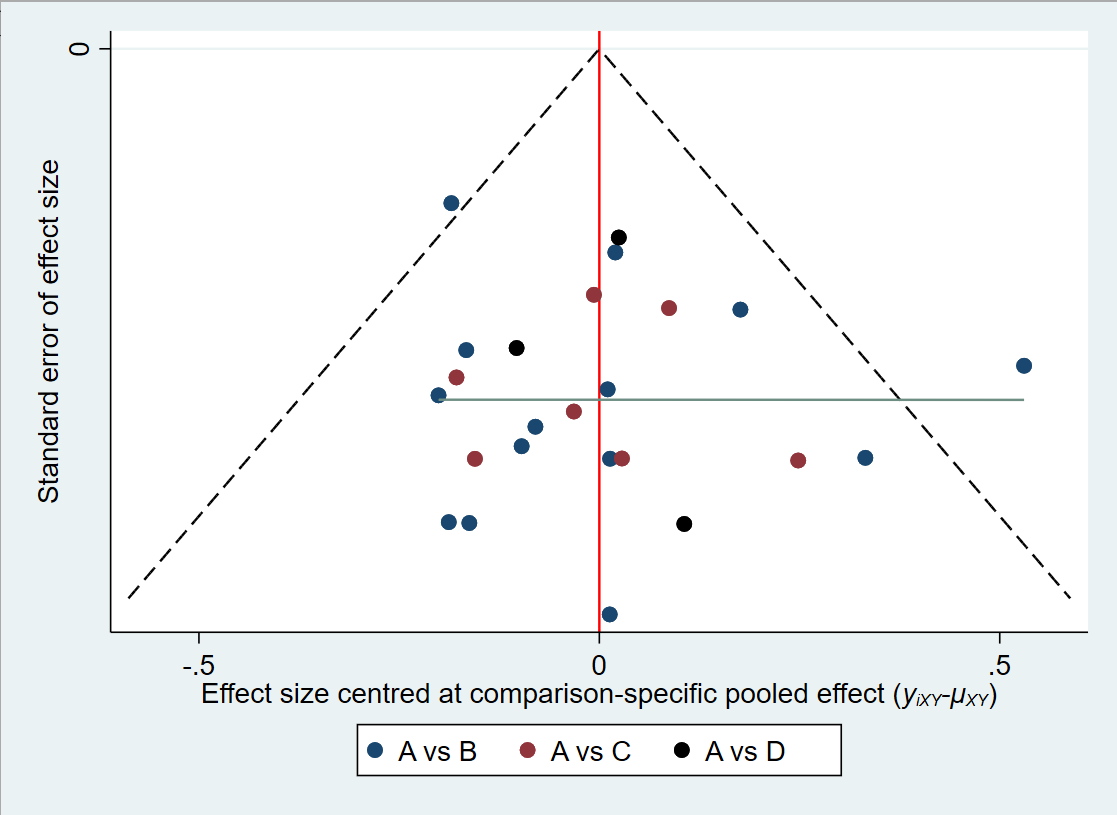


Fig. 37. Funnel plot of range of motion. A = conventional cutting instrument; B = computer-assisted navigation system; C = patient-specific instrument; D = robot-assisted system.


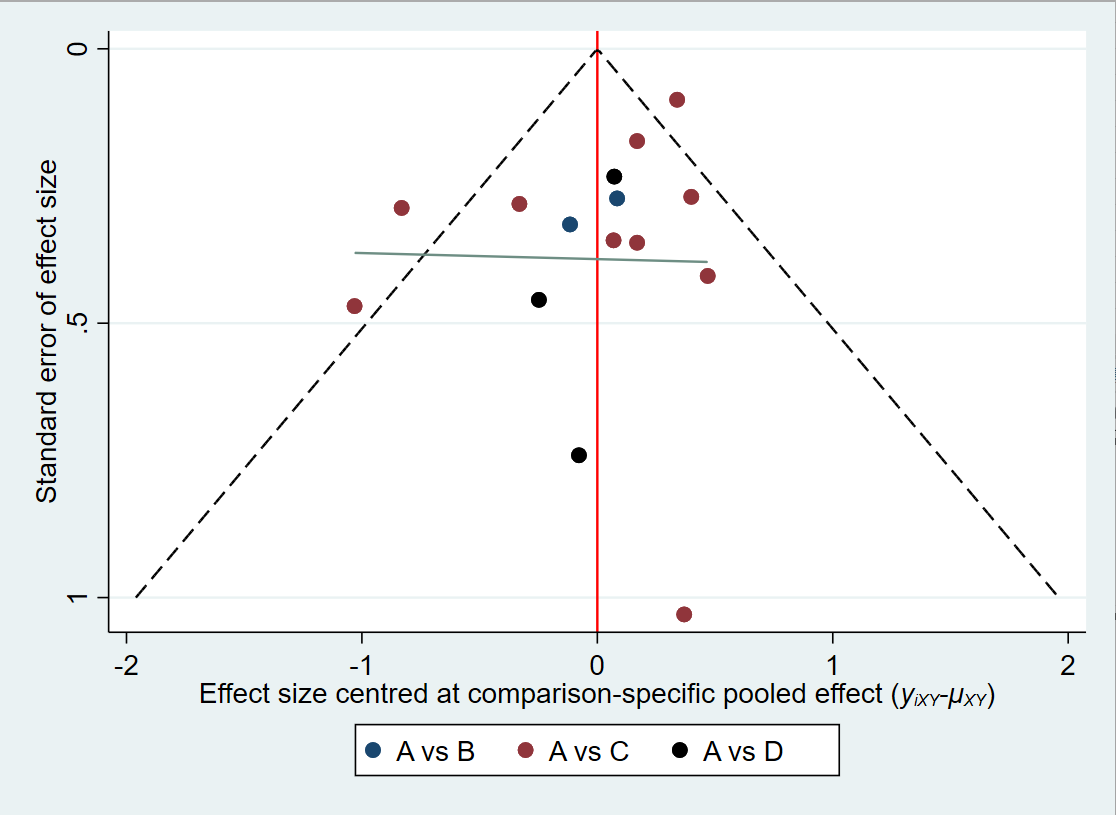


Fig. 38. Funnel plot of visual analogue scale scores. A = conventional cutting instrument; B = computer-assisted navigation system; C = patient-specific instrument; D = robot-assisted system.


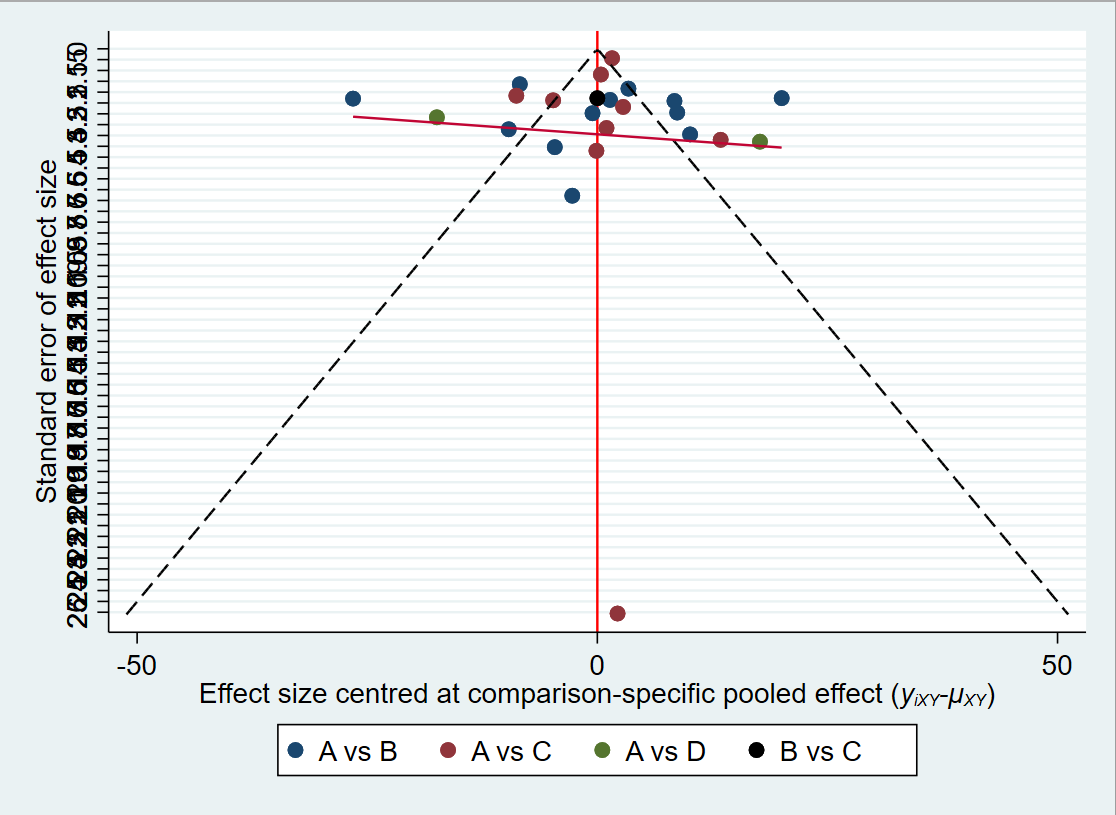


Fig. 39. Funnel plot of operative time. A = conventional cutting instrument; B = computer-assisted navigation system; C = patient-specific instrument; D = robot-assisted system.


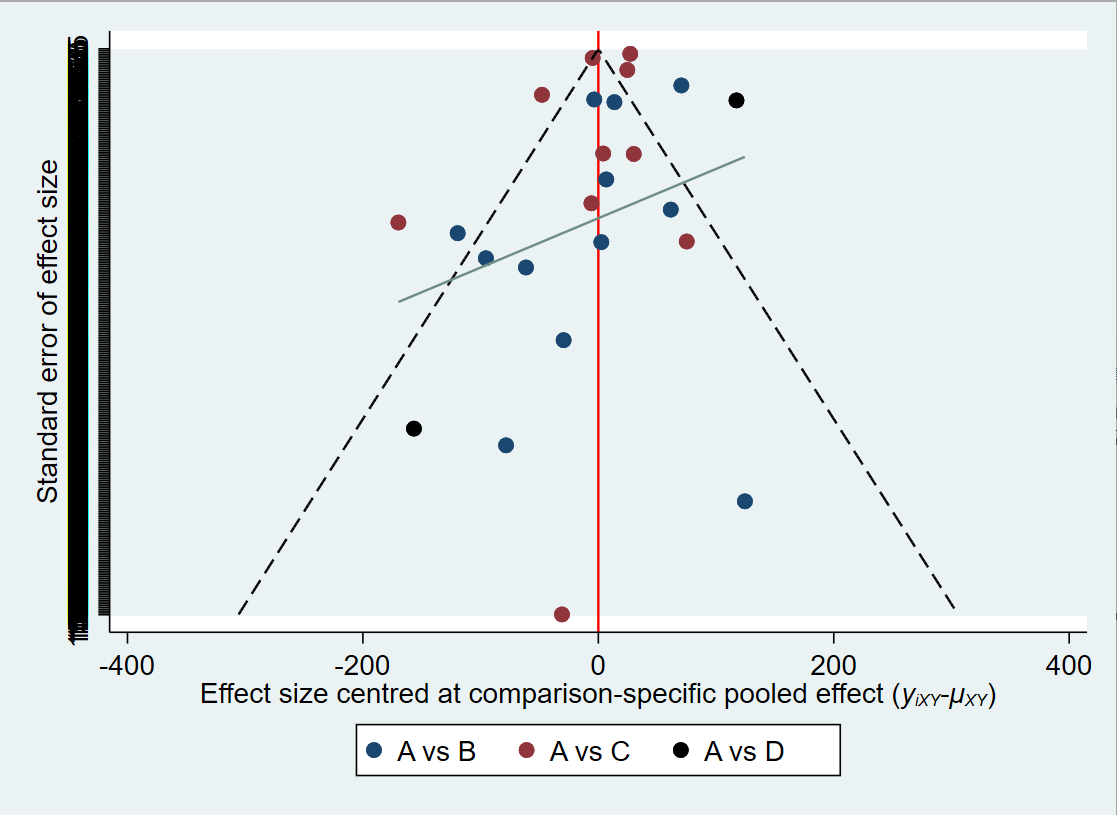


Fig. 40. Funnel plot of total blood loss. A = conventional cutting instrument; B = computer-assisted navigation system; C = patient-specific instrument; D = robot-assisted system.
